# Structure, Biology, and Therapeutic Application of Toxin–Antitoxin Systems in Pathogenic Bacteria

**DOI:** 10.3390/toxins8100305

**Published:** 2016-10-22

**Authors:** Ki-Young Lee, Bong-Jin Lee

**Affiliations:** Research Institute of Pharmaceutical Sciences, College of Pharmacy, Seoul National University, Seoul 151-742, Korea; kiyoung1983@snu.ac.kr

**Keywords:** toxin–antitoxin system, type II, structure–function correlation, pathogenic bacteria, novel antibiotic target

## Abstract

Bacterial toxin–antitoxin (TA) systems have received increasing attention for their diverse identities, structures, and functional implications in cell cycle arrest and survival against environmental stresses such as nutrient deficiency, antibiotic treatments, and immune system attacks. In this review, we describe the biological functions and the auto-regulatory mechanisms of six different types of TA systems, among which the type II TA system has been most extensively studied. The functions of type II toxins include mRNA/tRNA cleavage, gyrase/ribosome poison, and protein phosphorylation, which can be neutralized by their cognate antitoxins. We mainly explore the similar but divergent structures of type II TA proteins from 12 important pathogenic bacteria, including various aspects of protein–protein interactions. Accumulating knowledge about the structure–function correlation of TA systems from pathogenic bacteria has facilitated a novel strategy to develop antibiotic drugs that target specific pathogens. These molecules could increase the intrinsic activity of the toxin by artificially interfering with the intermolecular network of the TA systems.

## 1. Biological Roles of TA Systems

Recent advances in genome sequencing and bioinformatics have revealed a high prevalence of toxin–antitoxin (TA) systems in prokaryotes, which has served as a starting point for diverse and intensive studies of TA systems [1,2,3,4,5]. Over the past decades, a large number of divergent structures and physiological functions of proteins from bacterial TA systems have been characterized. TA systems are typically encoded in operons that are located on bacterial plasmids and chromosomes. The operons consist of adjacent toxin and antitoxin genes. Toxins have diverse cellular functions such as inhibitions of protein synthesis, DNA replication, and cell wall synthesis in response to unfavorable growth conditions. Many toxins are ribonucleases that degrade mRNA in a specific or nonspecific fashion, and some toxins act as gyrase inhibitors and kinases [6,7,8]. TA operons were first identified on the mini-F plasmid of *Escherichia coli* as important genetic elements responsible for cell survival; after cell division, only the daughter cell that harbours the TA genes on the transferable plasmid survives [9,10]. When a daughter cell does not have the parental plasmid that harbours these genes, the toxin kills the daughter cell because it is released from the residual non-toxic TA complex by the degradation of the cognate labile antitoxin. This is called post-segregational cell killing (PSK). In other words, the host becomes addicted to the continuous cellular expression of the antitoxin because it has a lower half-life than the toxin. The TA genes on the plasmid are often horizontally transferred, conferring antibiotic resistance and virulence in pathogenic bacteria. Additionally, the TA genes on chromosomes of various prokaryotes show alternative broad cellular functions that respond to diverse stressful environments such as nutrient deficiency, antibiotic treatment, bacteriophage infection, immune system attack, oxidative stress, and high temperature [11,12]. Depending on the types of TA systems, the activation of the toxin can eventually cause slow cell growth or cell cycle arrest, as is most frequently found in dormant persister cells [6,13,14,15]. Cell death, which is caused by excessive expression of the toxin, might benefit the maintenance of the whole population. A more widely accepted hypothesis is that the toxin is responsible for the cell arrest overcoming the temporary adversity. The hypothesis could be supported by the findings that later expression of the antitoxin can neutralize the virulence of the toxin and that organisms that are able to grow slowly or be dormant carry a large number of TA operons. For example, *Mycobacterium tuberculosis* harbours at least 30 functional TA operons covering five families of toxins, whereas its non-pathogenic counterpart, the faster-growing *Mycobacterium smegmatis*, has only three functional TA operons [16,17,18,19]. The dormant cell that has a low metabolic activity becomes partly or fully insensitive to the mechanism of action of antibiotic drugs, rendering many pathogenic bacteria resistant to multidrugs. This physiological state can switch back to a growing state once the drugs are eliminated. Therefore, this phenomenon, referred to as bacterial persistence, has been an obstacle to effective antibiotic treatment for many infectious diseases. The persistent cell is physiologically different from antibiotic-resistant mutants that proliferate in the presence of antibiotic drugs. It was initially proposed that in *Escherichia coli*, a high level of bacterial persistence is induced by high-persistence protein A (HipA) [20], which is later known to be encoded by the type II *hipBA* TA locus. HipA facilitates a transition to a persistent, antibiotic-insensitive cell by increasing the basal level of the synthesis of the secondary messenger (p)ppGpp [21], which is extensively distributed throughout bacteria. (p)ppGpp functions as an effector of the stringent response that induces the metabolism of the persistent state [22,23]. In *E. coli*, the persistence phenotype relies on (p)ppGpp, which activates type I and type II TA systems in a regulatory pathway involving the GTPase Obg, Lon protease, and polyphosphate [22,24,25]. (p)ppGpp inhibits the activity of exopolyphosphatases, causing polyphosphate to accumulate. The polyphosphate promotes the degradation of type II antitoxins by Lon protease, which releases toxins and thereby induces transient growth arrest. Obg-mediated persistence implicates the activation of TA systems; an increased level of Obg induces the expression of the type I toxin HokB in a (p)ppGpp-dependent fashion, leading to membrane depolarization and dormancy [24,25]. Another mechanism of persister formation is that the SOS response responsible for DNA repair induces the TisB toxin encoded by the type I TA locus *istR-tisB* [26]. The other 10 TA II loci of *E. coli*, encoding mRNases, contribute cumulatively to the establishment of persistent cells [27]. In *Salmonella enterica* serovar Typhimurium, the majority of the 14 TA modules are involved in the formation of non-replicating persister cells inside macrophages, which is responsible for chronic and recurrent infections [28]. This bacterium utilizes a distinct group of type I and type II TA systems to survive inside eukaryotic cells such as fibroblasts and epithelial cells [29].

Mutiple TA systems are related to the biofilm formation of human pathogenic bacteria, which empowers them to resist antibiotic drugs and host immune attacks [8,15,30,31]. Bacteria within biofilms show low growth rates, together with different expression profiles of specific genes in comparison to planktonic cells [32,33]. The MqsRA type II TA system is involved in biofilm formation via a complex regulatory gene network [34,35,36]. For example, MqsR is the motility quorum-sensing regulator that stimulates the expression of the transcription factor McbR [36,37]. McbR represses the production of periplasmic protein McbA, which prevents the synthesis of the exopolysaccharide colanic acid. Excess colanic acid provides mucoidy in biofilm formation. On the other hand, MqsA represses the expression of CsgD, the principle regulator of biofilm formation [35,38]. The inhibition of CsgD causes decreases in the signalling nucleotide c-di-GMP and curli production, which subsequently reduces biofilm formation. In addition, HipA releases extracellular DNAs that contribute to biofilm adhesion [39], and the TA modules of *E. coli*, YefM–YoeB, DinJ–YafQ, and RelBE, are related to biofilm formation in that they are activated by the overexpression of the biofilm inhibitor Hna [40].

## 2. Functions of Six Different Types of Bacterial TA Systems

Most toxins from TA systems modulate cell growth by binding to diverse important components of the cellular machinery. Functionally validated toxins of six different types of TA systems are listed in Table 1.

**Figure 1:**
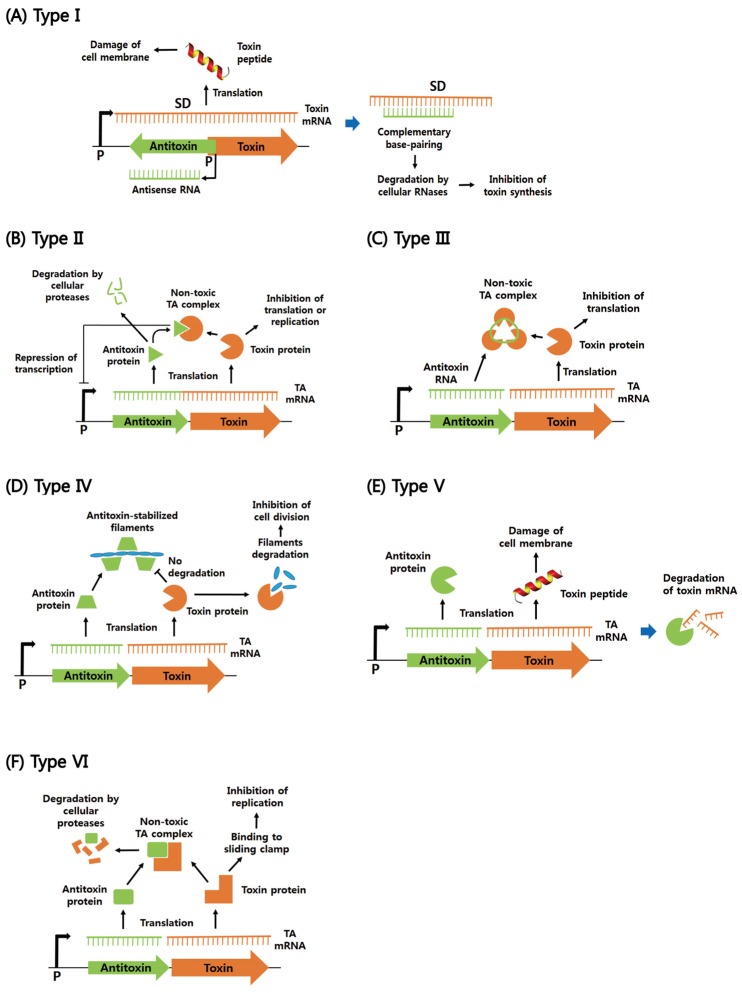
Schematic regulatory mechanisms of cellular functions of type I–IV' TA systems. (**A**) Type I TA system. Antisense RNA acts as an antisense that forms base pairs with toxin mRNA and thereby inhibits toxin synthesis. The toxin damages cell membrane, which inhibits essential cellular functions such as cell division and ATP synthesis. (**B**) Type II TA system. The toxin and antitoxin are proteins. Under normal growth conditions, the antitoxin inhibits the activity of the toxin by forming the TA complex. Commonly, the antitoxin alone or in complex with the toxin can bind to the TA promoter to repress transcription. Under unfavorable conditions, cellular proteases, such as Clp and Lon, are activated and degrade the antitoxins, which liberates the toxin to inhibit translation or replication. (**C**) Type III TA system. The antitoxin RNA binds to the toxin protein, forming the RNA pseudoknot–toxin complex. In the complex, the toxin is inactivated. (**D**) Type IV TA system. The protein antitoxin stabilizes filamentous cytoskeleton proteins (FtsZ and MreB), whereas the protein toxin destabilizes them and inhibits cell division. (**E**) Type V TA system. The protein antitoxin acts as a ribonuclease specific for the toxin mRNA. The peptide toxin is involved in membrane lysis. (**F**) Type VI TA system. The protein antitoxin behaves as a protease adaptor that delivers the protein toxin to a cellular protease, promoting its degradation. In the absence of the antitoxin, the toxin binds to the sliding clamp and inhibits DNA replication. Promoter and Shine–Dalgarno sequence are abbreviated as P and SD, respectively. Toxins and antitoxins are orange and green, respectively.

According to the mechanism by which the toxin is neutralized by the antitoxin, TA modules can be typically classified into six different types. Regulatory mechanisms of action of type I–VI TA systems are summarized in Figure 1.

### 2.1. Type I

Type I TA systems have a non-coding RNA antitoxin that complementarily binds to the toxin-encoding mRNA, which results in toxin mRNA degradation or inhibits toxin translation [74,75,76]. Type I toxin and antitoxin genes are independently transcribed from their own promoters, whereas other type TA operons are commonly co-transcribed from a single promoter upstream of the antitoxin gene. Most of type I toxins are small hydrophobic peptides that disrupt bacterial membrane integrity and thereby cause defects in membrane potential and cell division. They are predicted to have a conserved α-helical transmembrane domain for pore formation, but their cellular functions and action mechanisms are highly diverse [74,75,77]. It remains to be investigated whether the pore formation provokes a detergent-like effect, as is found in many antibacterial peptides [78].

Type I TA systems are not well characterized relative to type II TA systems, probably because current genomic techniques have not yet identified a large number of type I TA systems on plasmids and chromosomes. These systems were initially discovered on the par locus of plasmid pAD1 and the *sok-hok* pair of plasmid R1 [43,79]. The par system is the most extensively studied type I TA system, which has several homologues on plasmids and on chromosomes of Gram-positive bacteria [80]. The par locus on pAD1 can be transcribed into two small RNAs: toxin-encoding RNA I and antisense RNA II [81]. They form a complex by various complementary interactions. The *sok-hok* module participates in the extension of bacterial lag phase to adapt to stressful environments such as high temperature and antibiotic treatment [82]. The interaction between (p)ppGpp and Obg induces the expression of the Hok toxin through an unknown mechanism, which leads to membrane depolarization and a persistent state [24]. In addition, there are chromosomal type I TA systems in *E. coli* that include *ohsC-shoB* [46,83], *istR-tisB* [41], *rdlD-ldrD* [84], *agrB-dinQ* [85], *sibC-ibsC* [86], *orzO-zorO* [86], and *symR-symE* pairs [51]. Intriguingly, among these pairs, the DinQ and LdrD toxins of the *agrB-dinQ* and *rdlD-ldrD* systems can induce nucleoid condensation, and the SymE toxin of the *symR-symE* system lacks putative transmembrane domain and cleaves cellular mRNA. Similar to SymE, RalR encoded by the *ralA-ralR* type I TA locus of the *E. coli* rac prophage act as a non-specific endonuclease that cleaves methylated or non-methylated DNAs [87]. In *B. subtilis*, since the *ratA-txpA* pair was initially identified as the type I TA module located on the chromosome [48], four type I toxin families, TxpA/BsrG, BsrH/BsrE, YonT, and YhzE, have been proposed on the basis of sequence similarities between the different toxins of *B. subtilis* [88]. Most of the type I toxins from *B. subtilis* reside in prophage elements and are proposed to be required for prophage maintenance. In *Staphylococcus aureus*, the SprF1–SprG1 system was identified as an unconventional type I TA system that utilizes two initiation codons in a single reading frame to produce two SprG1 toxic peptides. The function of SprG1 is suppressed by SprF1, which is dual-acting antisense RNA [89]. Toxic peptides can be extracellularly secreted and then lyse human host erythrocytes and competing bacteria with different strengths [89]. Recently, a regulatory crosstalk between the type I TA system *ratA-txpA* and its adjacent type II TA system *mazEF* was discovered in the human pathogen *Enterococcus faecalis* [77]. The MazEF complex can not only function as an autorepressors but also increase transcription levels of antitoxin-encoding *ratA* by using different promoters. RatA decreases the stability of the toxin-encoding *txpA* mRNA.

### 2.2. Type II

Type II TA systems are the most widely distributed and the best characterized among prokaryotes and archaea. Such a system is commonly composed of toxin and antitoxin proteins that are expressed independently in the same operon but form a stable complex under normal growth condition, which renders the toxin post-translationally inactive [7,12,90,91,92]. In most cases, the TA complex or the antitoxin alone can bind to the corresponding TA operon and repress its transcription. A binding stoichiometry between toxin and antitoxin dictates the transcription level of TA operons in the cell, which can be explained by the mechanism of “conditional cooperativity”. Antitoxins commonly form dimeric proteins, each monomer is usually composed of the *N*-terminal domain required for dimerization and DNA binding and the *C*-terminal domain for toxin binding [93]. The catalytic site of the toxin is sterically blocked by the *C*-terminal region of the antitoxin. This domain is intrinsically disordered in a free state but typically undergoes coupled folding with toxin binding. Due to the flexibility of antitoxins, their entire structures have not been determined.

On the basis of structural and functional features of the toxin, bacterial type II TA modules can be roughly categorized into eight superfamilies: RelBE, MazEF, VapBC, CcdAB, ParDE, HigAB, HipBA, and Phd–Doc. Among them, the RelE, MazF, and HigB toxins are known as sequence-specific endo-ribonucleases that impede the global translations of cellular mRNAs [52,56,57,94,95,96,97,98,99,100]. The archaeal RelBE and *E. coli* MazEF families have been extensively studied. The RelE toxin is a ribosome-dependent ribonuclease that binds to the A-site of the ribosome and then cleaves nascent mRNA [57]. The RelB antitoxin wraps around the toxin, forming a large complex structure that inhibits the entrance of the toxin into the ribosomal A-site [101,102]. The MazEF systems were first identified as the ParDE and PemIK systems on plasmids R1 and R100, respectively, which are responsible for plasmid stability [103,104]. They are the first plasmid addiction systems to be identified, and the homologues were discovered on bacterial chromosomes. The MazF toxin cleaves mRNA specifically at ACA sequences [52], and its toxicity is abolished by binding to the MazE antitoxin. Recent data have shown that *E. coli* MazF specifically cleaves at an ACA triplet in 16S rRNA of 30S ribosomal subunit, leading to a loss of nucleotides in helix 45 and the anti-Shine–Dalgarno (aSD) sequence [53]. The modified ribosome with the rRNA truncation facilitates the selective translation of leaderless mRNAs produced by MazF. Two MazF toxins from *M. tuberculosis* target 23S or 16S rRNA at specific sites [105,106]; MazF-mt6 and MazF-mt3 cleave 23S rRNA at the evolutionarily conserved sequence UUCCU in the ribosomal A site [105,106], and MazF-mt3 also cleaves within the aSD sequence of 16S rRNA. MazF-mt9 from *M. tuberculosis* cleaves at a single site within the D-loop of tRNA^Pro14^ or the anticodon stem-loop (ASL) of tRNA^Lys43^ [107]. This high selectivity is conferred by recognition of sequence and structural determinants of tRNA. The CcdAB operon on the F plasmid is the oldest known TA module that couples plasmid replication to cell division, which was later described as a plasmid addiction system. The CcdAB complex functions as a repressor of its operon [108], but the transcription is in a dynamic equilibrium because the CcdA antitoxin is continuously degraded by the Lon protease [109]. The ternary complex of CcdB with the covalent gyrase-DNA complex leads to an inhibition of DNA transcription and to the accumulation of breaks in the chromosomal DNA [110]. CcdB also exerts its function by forming a binary complex with gyrase alone [111,112]. The inhibition of DNA gyrase mediates the SOS response and eventually causes cell death. The toxin–target complex can be dissociated by the CcdA antitoxin in a rejuvenation process [112]. Similar to CcdB, ParE blocks DNA replication by inhibiting the activity of DNA gyrase [113]. HipA acts as a serine/threonine kinase that phosphorylates glutamyl tRNA synthetase (GltX) and inhibits aminoacylation and translation, which are related to persister formation and multidrug tolerance [63,64]. Doc is a kinase that phosphorylates a highly conserved threonine (Thr-382) of the translation elongation factor EF-Tu, which hinders its binding to aminoacylated tRNAs and thereby inhibits protein synthesis [61,62]. *M. tuberculosis* VapC20 cleaves the well conserved Sarcin–Ricin loop (SRL) of 23S rRNA [59], which is the target of the eukaryotic toxins, α-Sarcin and Ricin [36,37]. A protein containing filamentation induced by cAMP (FIC) domain has recently been identified as a toxin of the FicTA type II TA family [114,115]. The toxin inhibits DNA replication by catalyzing the AMPylation of DNA gyrase and topoisomerase IV at their ATP binding sites. The molecular process induces cellular growth arrest and filamentation, which is believed to defense against phagocytosis of host immune cells [116].There are toxins that interfere with the association of ribosomal subunits and thus inhibit translation initiation. Ribosome association toxin A (RatA) from *E. coli* binds to the 50S subunit, blocks its interaction with the 30S subunit, and inhibits the formation of the 70S ribosome [117]. GraT of the GraTA system causes a defect in ribosome subunit assembly by a direct binding to the chaperone DnaK [118], which is known to be involved in late stages of ribosome biogenesis [119].

Many cellular targets and functions of the toxins could be fundamentally explained by even slight differences in their entire or local protein structures. The structures of two toxins, YoeB and Doc, are not homologous and use different mechanisms to inhibit protein synthesis. YoeB, which is structurally similar to RelE, has an RNase fold for interacting with the 50S ribosomal subunit [120]. The structure and function of HigB are similar to those of YoeB [56,121]. MazF is structurally similar to other family toxins such as Kid/PemK of the R100 plasmid and CcdB of the F plasmid [122]. Consistent with this similarity, the MazF and Kid toxins have similar functions as ribosome-independent, sequence-specific ribonucleases [123]. However, CcdB has a different function as an inhibitor of DNA gyrase, which is one example of structurally similar toxins having dramatically divergent cellular targets and functions. This case is also found in the RelE and CcdB families, which have similar protein folds but show completely different functions. Accumulating structural data on TA systems have revealed complex evolutionary relationships between them. Sequences and folds among toxins or antitoxins are not significantly conserved, and there exist various combinations of toxin and antitoxin folds. The YefM antitoxin of the YefM–YoeB system, which is one of TA modules in *E. coli*, is structurally similar to the Phd antitoxin of the Phd–Doc system of phage P1, although the sequence similarity between the two antitoxins is low [124]. YefM forms a heterotrimeric complex with YoeB in a similar manner to the Phd–Doc complex. Phd is structurally homologous to an antitoxin that binds to the toxin with an endonuclease activity, which is not related to Doc [125]. On the other hand, YoeB is structurally similar to the RelE toxin of the RelBE system. The observations suggest that antitoxin shuffling between different TA systems have evolutionally occurred.

### 2.3. Types III–VI

Type III TA system is composed of the protein toxin and the RNA antitoxin with multiple tandem repeats, which can form the non-toxic protein-RNA complex in a reversible manner [75,126]. The short palindromic repeat between toxin and antitoxin genes can form a transcriptional terminator, which controls the relative level of toxin mRNA and antitoxin RNA [69]. The toxin can function as an endoribonuclease that processes the antitoxin precursors into small RNAs and cleaves host RNAs. In addition, this system plays an important role in abortive phage infection (abi) by degrading phage RNAs, which promotes altruistic suicide of phage-infected cells. The *toxIN* pair located on a plasmid from *Pectobacterium atrosepticum* was first identified as type III TA system [69]. The antitoxin ToxI folds into an RNA pseudoknot, and its full-length sequence (200 nt) is composed of 5.5 sequence repeats of 36 nt, but even a single repeat unit is able to annul ToxN toxicity. The crystal structure of the ToxIN complex showed a triangular heterohexamer which consists of three ToxN toxins and three ToxI RNA pseudoknots [127]. Another known type III TA system is the *antiQ-abiQ* pair on a plasmid of *Lactococcus lactis*, which is structurally and functionally similar to the *toxIN* pair although their gene sequences show only 31% similarity [128]. In type IV TA system, the protein antitoxin does not bind directly to the toxin but instead competes for the same cellular target, which indirectly antagonizes toxin function [70]. For example, the *E. coli* TA modules *yeeUV* and *cptBA* encode the toxins YeeV and CptA, respectively, which block the polymerization of bacterial cytoskeletal proteins MreB and FtsZ. The corresponding antitoxins YeeU and CptB counteract the toxicity by stabilizing the filamentous polymers [70]. Interestingly, YeeU has a similar fold to those of the YoeB and RelE toxins [125], suggesting evolutionary exchange between toxin and antitoxin families. Type V TA system has the protein antitoxin that degrades mRNAs of the corresponding toxin, suppressing the cellular expression of the toxin. This system was exemplified by the GhoST module encoding the small peptide toxin GhoT that damages the cell membrane and the antitoxin GhoS that cleaves the toxin mRNA [72]. Interestingly, under unfavorable conditions, the type II toxin MqsR cleaves the GhoS mRNA, thus promoting the translation of GhoT [129]. The *socAB* operon was most recently identified as a type VI TA system [73]. The protein toxin SocB binds tightly to the β sliding clamp DnaN and thus represses replication elongation, and the antitoxin SocA behaves as a proteolytic adaptor protein to bind the toxin, which expedites protease-mediated degradation of the toxin.

## 3. TA Modules from Pathogenic Bacteria

Human pathogenic bacteria have evolved many TA systems to protect themselves from stressful circumstances encountered in the host cell. A comprehensive bioinformatics search has recently revealed a large number of type II TA operons in bacteria [1], and much effort has been paid to their structural and functional validations. On the basis of the TA bioinformatics, type II TA pairs from important human pathogenic bacteria are listed in Supplementary Table S1.

### 3.1. Gram-Positive Bacteria

*Streptococcus pyogenes* is the causative agent of many human diseases such as superficial skin infections, pharyngitis, erysipelas, and cellulitis. Several type II TA families are extensively distributed throughout *S. pyogenes* species: five HicBA, five MazEF, two omega–epsilon–zeta (ω–ε–ζ), seven ParDE, and 19 RelBE pairs (see Supplementary Table S1). In particular, ω–ε–ζ TA module was discovered as a PSK system on plasmid pSM19035 from *S. pyogenes* [130]. The ω–ε–ζ system not only stabilizes resistance plasmids but also promotes virulence and is transcriptionally modulated by another repressor protein designated ω [131,132]. The ε antitoxin acts solely as an inhibitor of the ζ toxin, not a transcription repressor, which distinguishes it from many other antitoxins. When the ε antitoxin is continuously degraded by AAA+ proteases and not replenished by protein synthesis, the ζ toxin dissociates with it and eventually causes cell death [133]. *Streptococcus pneumoniae* is a significant human pathogenic bacterium that asymptomatically infects the respiratory tract and becomes pathogenic in immune-compromised individuals [134]. This bacterium has been recognized as the main cause of community acquired pneumonia and meningitis in children and the elderly. *S. pneumoniae* species harbour type II TA pairs including five HicBA, one ω–ε–ζ, one PezAT, four Phd-Doc, and 17 RelBE (see Supplementary Table S1). There are four functionally well-characterized type II TA operons, namely *relBE2*, *pezAT*, *yefM-yoeB*, and *phd-doc*, and several putative TA operons depending on the strains [4,135]. The pneumococcal RelE2 toxin shows a similar toxicity for both *S. pneumoniae* and *E. coli*, but its cognate antitoxin RelB2 seems to neutralize the toxicity in a certain growth time [136], which contrasts with those of the *E. coli* RelB antitoxins. In solution state, RelB2 alone is present as a dimer, but the RelBE complex is formed predominantly in a ReB2:RelE2 stoichiometry of 4:2 [137]. This heterohexamer can bind to one operator DNA molecule. In contrast, the *E. coli* RelBE complex is assumed to exist as a heterotetramer or a heterotrimer in solution [97,138]. RelE1, one of putative toxins of *S. pneumoniae*, has no RNase activity [57] and is not harmful to both *S. pneumoniae* and *E. coli* [135]. The pneumococcal YoeB toxin was reported to inhibit the cellular growth of *E. coli* [139]. The toxicity is much lower in a Lon protease-deficient strain than in the wild-type strain [139], possibly because Lon protease degrades the cognate antitoxin YefM and thus increases the toxicity of YoeB. Similar to other type II antitoxins, YefM is thermally less stable than the YefM–YoeB complex, which was demonstrated by a lower melting temperature of YefM (~45 °C) [139]. The *phd-doc* pair is most recently identified as the fourth *bona fide* TA operon in *S. pneumoniae*, showing typical features of type II TA systems [135]; (i) overexpression of the Doc toxin strongly inhibits the growth of *S. pneumoniae*, which is resumed by co-expression with the Phd antitoxin; and (ii) the *phd-doc* promoter is slightly and almost completely repressed by Phd and Phd–Doc complex, respectively. The PezAT TA system located on the chromosome of *S. pneumoniae* is another version of the pneumococcal plasmid-encoded ε–ζ system. PezT inhibits the cell growth of *E. coli* for the first 3 h, which is subsequently resuscitated without the need for co-expression with the PezA antitoxin [140]. This growth profile is similar to the profile yielded by the ζ toxin [141]. The toxicity of PezT can be neutralized by the antitoxin PezA, which is a multi-domain protein. PezAT modules located on pneumococcal pathogenicity islands could provide the host with virulence factors and antibiotic resistance [142,143]. *E. faecalis* is a human commensal bacterium that causes endocarditis, septicaemia, urinary tract infections, and meningitis while inhabiting the human gastrointestinal tract [134,144]. This bacterium is known to have type II TA pairs including two MazEF, one Phd–Doc, and two RelBE (see Supplementary Table S1). In addition, the par addiction operon of the plasmid pAD1 of *E. faecalis* encodes the hydrophobic Fst toxin consisting of 34 residues and a small antisense RNA that complementarily binds to the toxin mRNA, which is characteristic of type I TA system. Fst has neither haemolytic nor antimicrobial activities [145], strengthening the possibility that it has negative effects on chromosome segregation and cell division. *S. aureus* commonly causes skin and respiratory diseases such as abscesses, impetigo, sinusitis, and pneumonia [146]. The emergence of methicillin-resistant *S. aureus* (MRSA) has become a worldwide problem in the 21st century [147]. The *S. aureus* genome contains three main type II TA operons, namely *mazEF*, *yefM-yoeB*, and ω-ε-ζ, among which *mazEF* has been most extensively studied [5]. Multiple *Bacillus* species are considered important Gram-positive human pathogens; for example, *B. anthracis* causes anthrax, and *B. cereus* causes food poisoning. The TA database has suggested that *B. anthracis* and *B. cereus* have two and three MazEF TA systems, respectively. Non-pathogenic *B. subtilis* has proved an excellent model for molecular and cellular biology research [134]. The SpoIISA–SpoIISB operon encoded on *B. subtilis* chromosome was initially shown to be a novel TA system in a screen for *B. subtilis* mutants that are defective in sporulation after the formation of the polar septum [148]. The SpoIISA toxin inhibits the formation of cell envelopes after asymmetric septation.

### 3.2. Gram-Negative Bacteria

*Neisseria gonorrhoeae* is the causative agent of the sexually transmitted disease, gonorrhea [149]. After survival inside epithelial cells, *N. gonorrhoeae* can penetrate the epithelial monolayer to infect the stromal tissue of the subepithelium, giving rise to purulent inflammation characteristic of gonorrhoea [150,151]. A bioinformatics search has revealed that *N. gonorrhoeae* species have type II TA pairs including two HicBA, two MazEF, three RelBE, and two VapBC (see Supplementary Table S1). In addition, the FitAB TA system was identified through genetic screens, not bioinformatics [149]. This system could facilitate a dormant state of the pathogen within epithelial cells, which circumvents human immune attacks. This state also allows the host to be an asymptomatic carrier, promoting the spread of the disease. FitAB deletion causes *N. gonorrhoeae* to grow faster than wild-type inside host cells. *R. felis* is a flea-borne pathogenic bacterium that causes spotted fever in humans [152]. The *R. felis* genome harbours at least 13 TA systems predicted to act as addiction systems [153]. There exist type II TA pairs in *R. felis* organisms: one HicBA, one MazEF, 12 RelBE, and six VapBC (see Supplementary Table S1). Among these, the VapC toxin was shown to exhibit a ribonuclease activity and thereby inhibits the growth of *E. coli* and *Saccharomyces cerevisiae* [154]. *Shigella flexneri* is a pathogenic bacterium that mainly causes diarrhoea in humans [134]. This pathogen has type II TA pairs including one VapBC, one MazEF, one ParDE, and six RelBE (see Supplementary Table S1). VapC (MvpT) toxin encoded on virulence plasmid pMYSH6000 cleaves the initiator tRNA^fMet^ specifically in the anticodon region and therefore inhibits the global cellular translation [58], which is similar to the function of VapC toxins from enteric bacteria [58]. In addition, in the YeeUW(V) system from *S. flexneri*, the YeeU antitoxin represses the expression of the YeeV toxin without direct physical interaction with it [70]. The action mechanism of this system is fundamentally different from those of other well-known bacterial TA systems. *E. coli* O157 is a human enterohemorrhagic pathogen that causes haemorrhagic diarrhoea and kidney failure [155]. *E. coli* O157 infection occurs primarily through consumption of contaminated and raw foods [156]. The *E. coli* O157 genome harbours type II TA pairs including two CcdAB, two HicBA, two HigBA, five MazEF, two ParDE, and nine RelBE (see Supplementary Table S1). There are two three-component TA operons related to the parDE system, the EcPaaR1–EcPaaA1–EcParE1 and EcPaaR2–EcPaaA2–EcParE2 modules on the prophage region [157]. A three-component TA system is also observed for the ω–ε–ζ module on the plasmid of *B. subtilis* [158]. In contrast to typical type II TA systems, the antitoxin of the three-component system lacks a DNA-binding domain, which is instead adopted by a third protein that acts as transcriptional repressor of the TA operon. The DNA-binding domain of EcPaaR1 and EcPaaR2 belongs to the DicA transcriptional repressor family. *S*. *typhimurium* is a foodborne pathogen that causes inflammatory disease in the gastrointestinal tract of humans [134]. After penetrating the intestinal barrier and spreading via the lymphatic and hematogenous routes, this bacterium settles in the spleen, bone marrow, Peyer’s patches, liver, bile ducts, etc., causing typhoid fever. The TA database has suggested that *S*. *typhimurium* genome harbours type II TA pairs including three HigBA, three Phd–Doc, 13 RelBE, and five VapBC (see Supplementary Table S1). *Salmonella* species can be transformed into non-replicating persister cells inside macrophages, which are involved in at least 14 putative type II TA families [28]. Three of the 14 TA families have toxin components that share sequence similarity with Gcn5 N-acetyltransferase (GNAT) enzymes, and one of the three toxins, named TacT, was structurally and mechanistically characterized [159]. TacT acetylates aminoacyl-tRNA molecules and thereby inhibits protein translation, promoting the formation of persister cells. This activity is well consistent with its crystal structure showing an acetyltransferase fold in a complex with Ac-CoA molecule. *B. abortus* naturally infects ruminants. The infection of humans is zoonotic and causes a debilitating chronic disease known as undulant fever by primarily residing in phagocytic cells and placental trophoblasts [160]. This bacterium is predicted to have two classified type II TA operons, Phd–Doc and RelBE (see Supplementary Table S1). *Vibrio* species can cause gastroenteric disorders such as diarrhoea, abdominal cramps, nausea, vomiting, etc., which commonly occurs after consumption of contaminated seafood or exposure of wounds to contaminated seawater [134]. Pathogenic *Vibrio* species include *V. cholerae*, *V. parahaemolyticus*, and *V. vulnificus*. A bioinformatics search has revealed type II TA pairs throughout *Vibrio* pathogens: two HigBA, one HipBA, three ParDE, 13 RelBE, one CcdAB in *V. cholerae*; one HipBA, two ParDE, four RelBE, and five CcdAB in *V. parahaemolyticus*; and one HicBA, three HipBA, one ParDE, five RelBE, and two CcdAB in *V. vulnificus* (see Supplementary Table S1).

### 3.3. Mycobacterium Tuberculosis

*M. tuberculosis* is neither Gram-positive nor Gram-negative and instead classified as an acid-fast bacterium. This bacterium lies dormant inside macrophages in the human respiratory system and has the potential to cause tuberculosis [134]. *M. tuberculosis* H37Rv possesses a total of 79 chromosomal TA systems, which can be categorized into 68 well-known and 11 putative TA systems [3]. There are six well-described type II TA families, including VapBC (50 systems), MazEF (10 systems), RelBE (two systems), HigBA (two systems), ParDE (two systems), and YefM/YoeB (one system). Among these, some VapBC systems have been biochemically well studied to date. For example, fluorescence experiments using fluorescent-labelled RNA substrates of unknown sequence revealed that two VapC toxins, Rv0624 and Rv0627, have ribonuclease activities that are dependent on Mg^2+^/Mn^2+^ and Mg^2+^ ions, respectively. Rv0624 can also degrade tRNA^fMet^, though its activity is relatively low. The VapC2 toxin (Rv0301) has a ribonuclease activity that cleaves single-stranded viral MS2 RNA [18]. However, the catalytic mechanism involving divalent ions is not well characterized.

## 4. Structural Characteristics of TA Proteins from Pathogenic Bacteria

In this section, in order to provide valuable information for the structure-based design of novel antibiotics that modulate the TA systems, we mainly explore the well-known structures and interaction modes of TA proteins from 12 important pathogenic bacteria. Notably, the type II TA families, MazEF, CcdB, and YefM-YoeB, with known structures from non-pathogenic *E. coli* K12 are also found in pathogenic *E. coli* O157. The TA proteins described in this section, except for *E. faecalis* Fst toxin, belong to type II TA families. TA proteins with known structures from pathogenic bacteria are listed in Table 2.

### 4.1. MazEF

**Figure 2:**
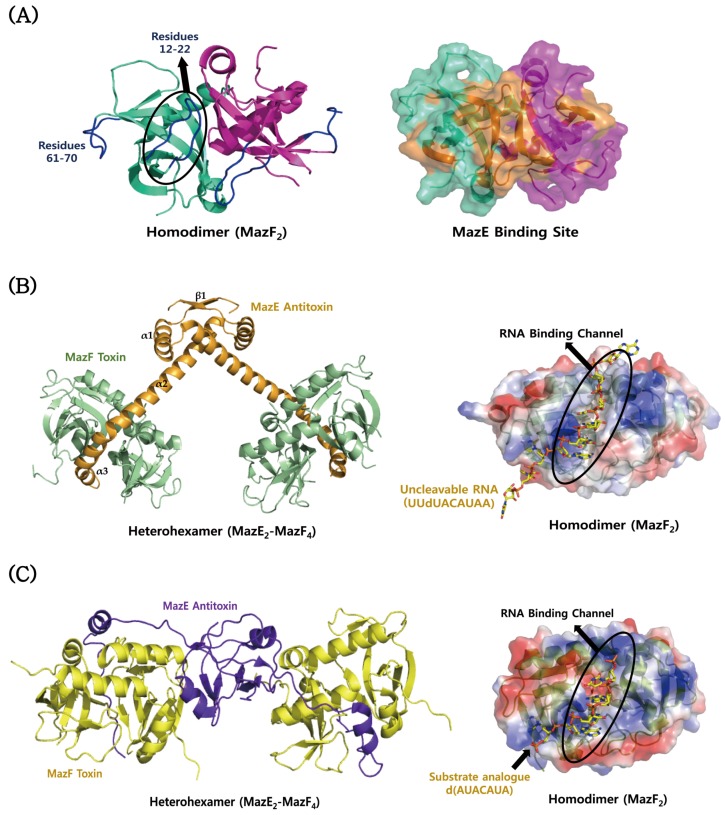
Structural comparisons of MazEF proteins from *S. aureus*, *B. subtilis*, and *E. coli*. (**A**) Structure of *S. aureus* MazF. The residues showing a relatively high mobility are indicated in blue. MazE binding site is mapped in orange onto the surface model of MazF. (**B**) Structure of *B. subtilis* MazF in complex with *B. subtilis* MazE (left panel) and its uncleavable RNA substrate (UUdUACAUAA) (right panel); (**C**) structure of *E. coli* MazF in complex with *E. coli* MazE (left panel) and the substrate analogue [d(AUACAUA)] (right panel). In (**B**) and (**C**), an RNA binding channel of MazF is formed by its dimerization.

The crystal structure of the MazF toxin from *S. aureus* shows a well-defined dimer, each monomer consisting of a core five-stranded anti-parallel β-sheet surrounded by three α-helices and a small two-stranded anti-parallel β-sheet (Figure 2A) [161], which is featured by the typical MazF/CcdB fold. Crystallographic and nuclear magnetic resonance (NMR) studies revealed structural and dynamics differences in three loop regions of the MazF toxin, two of which are relevant to the function [161]. The two loops, including residues Leu12–Gly22 and Ile61–Lys70, show a relatively low heteronuclear nuclear Overhauser effect (hetNOE) values and a decrease in the ratio of the transverse relaxation rate (R2) to the longitudinal relaxation rate (R1) indicative of increased mobility in the fast NMR timescale. NMR titration experiments showed that a MazE-derived peptide consisting of residues Met23–Glu56 causes significant chemical shift changes in loop 1 and strands S5 and S6 of MazF [161], suggesting that these regions are involved in MazE binding. The residues with a high mobility and the MazE binding site are colour-mapped onto the structure of MazF in Figure 2A. *S. aureus* MazF is structurally most similar to *B. subtilis* MazF: their structures show an RMSD value of 0.73 Å for 110 Cα atoms. The structural difference is mainly found in the loop Gly48–Ile55 of *S. aureus* MazF, which corresponds to the loop Ala48–Leu55 of *B. subtilis* MazF. Another structural homologue of *S. aureus* MazF is *E. coli* MazF: their structures show an RMSD value of 1.69 Å for 95 Cα atoms. *S. aureus* MazF adopts different conformations in the loops Gly48–Ile55 and Leu9–Pro25 and the helix Ile29–Thr40, compared to *E. coli* MazF. NMR dynamics studies showed that they share three loop regions with a relatively high flexibility: S1-S2, S3-S4 and S4-S5 [161]. In *B. subtilis*, there are the known structures of MazF bound to MazE and uncleavable UUdUACAUAA RNA substrate [179]. The TA proteins form a heterohexameric complex with a stoichiometry of two MazF monomers per MazE (Figure 2B). The two subunits of MazF form an extensive dimeric interface with a concave surface. The overall structure of *B. subtilis* MazF is highly similar to that of *E. coli* MazF. The *N*-terminal domain of MazE contains β1 strand and α1 helix followed by the *N*-terminal region of α2 helix. This domain is responsible for dimerization, which forms a RHH motif for DNA binding. On the other hand, the *C*-terminal domain of MazE contains the *C*-terminal region of α2 helix and the short α3 helix, which bind tightly to a cleft formed by the dimerization of MazF. MazE prevents an RNase activity of MazF by sterically blocking the RNA binding channel of MazF. In contrast to the structural features of *B. subtilis* MazE, *E. coli* MazE contains the *N*-terminal β barrel core for DNA binding and the *C*-terminal long loop for MazF binding (Figure 2C). Consistent with the structural difference, *E. coli* MazE cannot inhibit the toxicity of *B. subtilis* MazF [180]. The MazF structures in complex with uncleavable substrate analogues from *B. subtilis* and *E. coli* are shown in Figure 2B,C. The structural comparison reveals that they have a common mode of substrate recognition on a large interaction surface [167]. However, the substrate binding of *E. coli* MazF is achieved by fewer hydrogen bonds to specific RNA bases and poorer surface complementarity than that of *B. subtilis* MazF, possibly explaining the relaxed substrate specificity of *E. coli* MazF.

### 4.2. CcdAB

**Figure 3:**
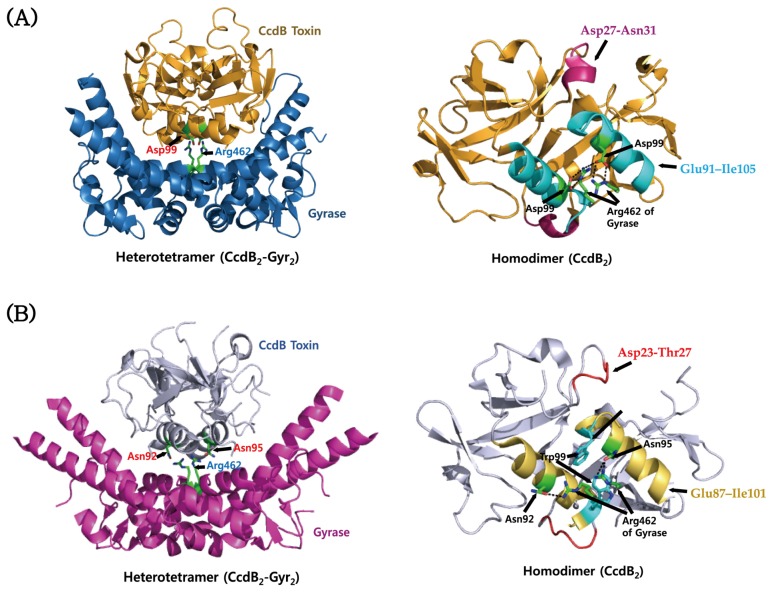
Structures of the CcdB–gyrase complexes from *V. fischeri* and *E. coli*. (**A**) Structure of the CcdB–gyrase complex from *V. fischeri* (left panel) and the gyrase binding site of the CcdB dimer (right panel). Gyrase binding segments include the residues Asp27–Asn31 and Glu91–Ile105, which are marked in purple and cyan, respectively. Two residues, Asp99 and Arg462, forming the hydrogen bond between CcdB and gyrase are indicated. (**B**) Structure of the CcdB–gyrase complex from *E. coli* (left panel) and the gyrase binding site of the CcdB dimer (right panel). Gyrase binding segments include the residues Asp23–Thr27 and Glu87–Ile101, which are marked in red and dark yellow, respectively. Trp99, which is involved in gyrase binding, is marked in cyan. Three residues, Asn92, Asn95, and Arg462, forming the hydrogen bonds between CcdB and gyrase are indicated. Hydrogen bonds are depicted as dotted lines.

The CcdB toxin from *V. fischeri* shows a high sequence similarity (~80%) to those from pathogenic *Vibrio* species, suggesting that they share highly conserved structures. The crystal structure of the CcdB–Gyrase complex from *V. fischeri* was revealed as shown in Figure 3A. This protein can form a homodimer in which a relatively rigid dimeric interface is created by a hydrophobic core, and its individual monomers can rotate relative to each other due to their structural flexibility [170]. Binding stoichiometries of CcdA_2_–CcdB_4_ and CcdA_2n_–CcdB_2n_ are responsible for the neutralization of the toxicity and the down-regulation of its operon. In solution, the dimeric CcdB toxin can unfold into monomeric components, which have a partial secondary structure. There exist significant structural differences between the CcdB toxins from the *V. fischeri* chromosome and the *E. Coli* F plasmid, suggesting that they belong to different families. The chromosomal toxin has the CcdA- and gyrase-binding sites which have many non-conserved residues compared to the F plasmid toxin. In agreement with this, the two CcdB toxins show different affinities for their corresponding CcdA antitoxins and gyrases [171], suggesting the possibility that TA modules of plasmids and chromosomes play different cellular roles. The CcdB–gyrase complexes from *V. fischeri* and *E. coli* exhibit similar overall architectures [169,171] (Figure 3). However, the region Asp27–Asn31 of *V. fischeri* CcdB adopts different conformation from the corresponding region Asp23–Thr27 of *E. coli* CcdB, which causes different interactions with gyrases. In addition, the gyrase binding mode involving the α-helix region Glu91–Ile105 of *V. fischeri* CcdB differs from the corresponding region Glu87–Ile101 of *E. coli* CcdB. Among these regions, only three out of 10 residues that interact with gyrase are conserved between them. In the CcdB–gyrase complex from *V. fischeri*, Arg462 of gyrase forms two hydrogen bonds to Asp99 of CcdB (Figrue 3A). In the CcdB–gyrase complex from *E. coli*, there exist two different conformations for the side chains of Arg462, which form hydrogen bonds with Asn95 and Asn92, respectively (Figure 3B). The latter residue is replaced by Ala96 in *V. fischeri* CcdB, which does not make a hydrogen bond to Arg462. Interestingly, in contrast to *V. fischeri* CcdB, *E. coli* CcdB undergoes a structural rearrangement of Trp99 upon gyrase binding such that its buried sidechain is exposed toward Arg462 of gyrase.

### 4.3. VapBC

**Figure 4:**
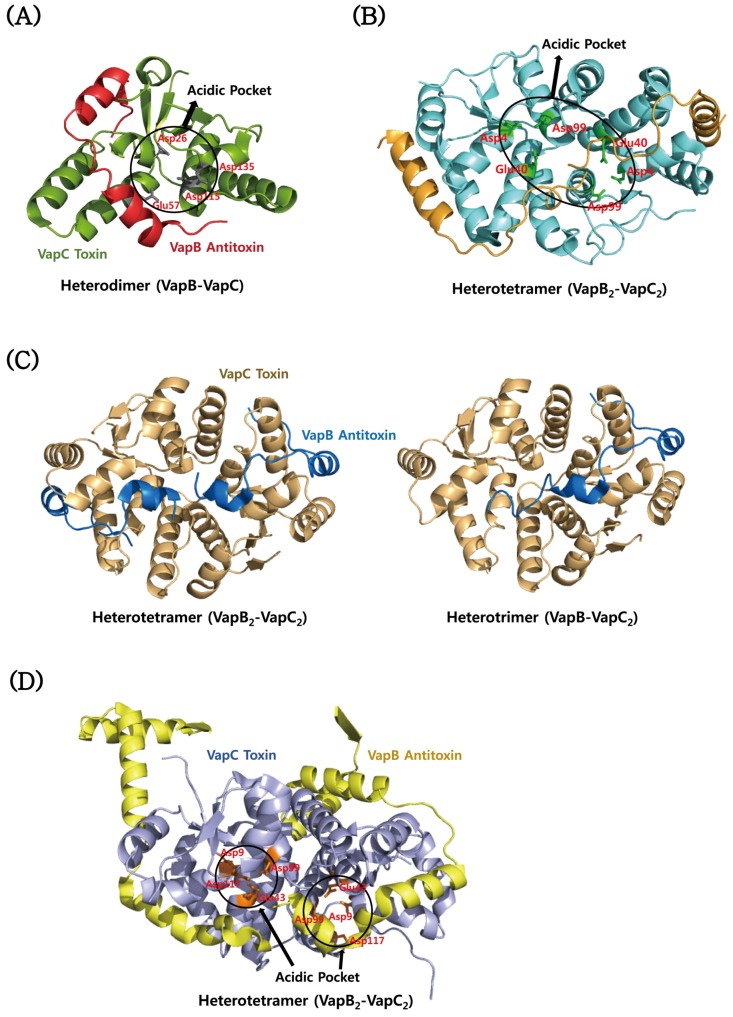
Structural comparisons of VapBC proteins from *M. tuberculosis.* (**A**) Structure of the VapBC complex (Rv0626–Rv0627). Four conserved residues of VapC are clustered to form an acidic pocket. (**B**) Structure of the VapBC complex (Rv0623–Rv0624). Three conserved acidic residues of each VapC subunit are positioned in the dimeric interface of VapC. (**C**) Structure of the VapBC complex (Rv2009–Rv2010). VapC dimers bind to one and two VapB with a stoichiometry of 2:1 and 2:2, respectively. (**D**) Structure of the VapBC complex (Rv0300–Rv0301). Four conserved residues of each VapC monomer are clustered to form an acidic pocket.

**Figure 5:**
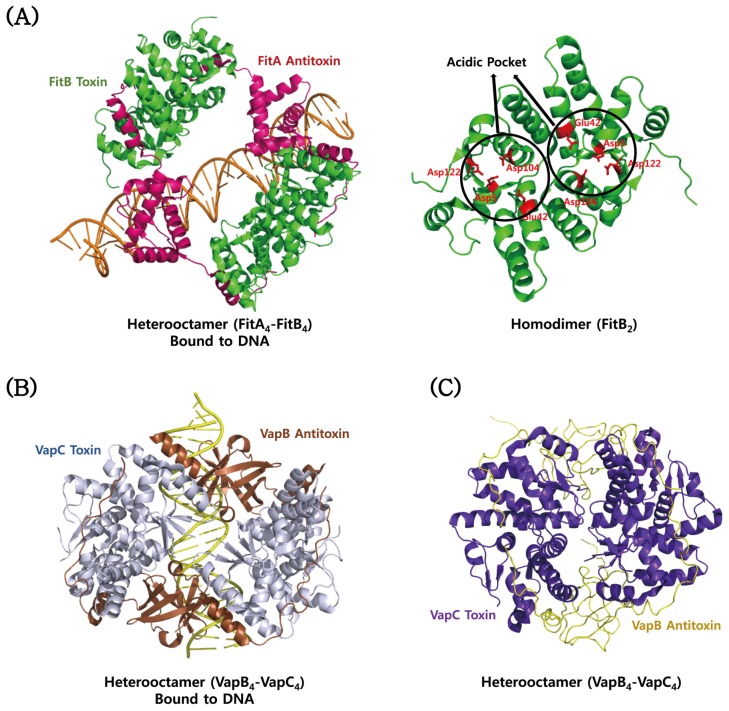
Structural comparisons of FitAB and VapBC proteins from *N. gonorrhoeae*, *R. felis*, and *S. flexneri*. (**A**) Structure of the FitAB–DNA complex from *N. gonorrhoeae*. The conserved acidic residues that form an acidic pocket of the FitB toxin are indicated on the right side. (**B**) Structure of the VapBC–DNA complex from *R. felis*; (**C**) structure of the VapBC complex from *S. flexneri*.

The VapC toxins belong to the pilT *N*-terminal domain (PIN-domain) family, which share an α/β/α sandwich structures and four conserved catalytic acidic residues although sequence identity within them is low [181]. The structures are highly similar to those of the T4 RNase H nuclease domain [17]. The structure of *M. tuberculosis* VapC toxin (Rv0627) in complex with the *C*-terminal region of its cognate VapB antitoxin (Rv0626) was determined [173]. The structure of Rv0627 shows a compact α/β/α core domain and a protruding clip structure of two α-helices (Figure 4A). The core domain is composed of a four-stranded parallel β-sheet that is surrounded by five α-helices. The structure of the bound Rv0626 antitoxin shows 33 out of 86 residues. The missing residues are located in the *N*-terminal DNA binding region of the antitoxin. The TA complex is achieved mainly by hydrophobic interactions in a groove formed between the core domain and the clip structure of the toxin. The VapC toxin has four highly conserved acidic residues, Asp26, Glu57, Asp115, and Asp135, which are clustered to form a negatively charged cavity (Figure 4A). The crystal structure of the VapBC complex (Rv0623–Rv0624) from *M. tuberculosis* revealed distinct structural differences between the Rv0624 toxin and PIN domain proteins, although they have similar overall architectures [176] (Figure 4B). The *C*-terminal fragment of Rv0624 does not form the secondary structure, whereas the corresponding region of other VapC members commonly forms an α-helix followed by one β-strand. In addition, Rv0623, except for the *N*-terminal α-helix, is structurally different from other VapB members, and its interaction with Rv0624 shows a characteristic structural feature. Three conserved residues (Asp4, Glu40 and Asp99) of the active site of the toxin are positioned in a concave surface of the homodimeric interface of the toxin (Figure 4B). These residues coordinate a Mg^2+^ ion by a hydrogen bond network. The crystal structure of the VapBC complex (Rv2009–Rv2010) from *M. tuberculosis* revealed one tetramer of VapBC heterodimers and two heterotrimers (VapBC_2_) in each asymmetric unit of the crystal (Figure 4C) [175]. This observation indicates that the VapB antitoxin can bind to the VapC toxin in both 1:1 and 1:2 ratios. The catalytic site of the toxin consists of spatially close acidic residues and two divalent metal ions. High-resolution crystal structure of the VapBC complex (Rv0300–Rv0301) from *M. tuberculosis* shows Mg^2+^ ions that are bound and unbound to the active site of the Rv0301 toxin, which forms a tightly packed globular protein with the characteristics of a typical PIN-domain (Figure 4D) [174]. The conserved acidic residues of the toxin are Asp9, Glu43, Asp99, and Asp117. The Rv0300 antitoxin adopts significantly different conformations due to its different hinges, called open and closed antitoxins, which are wrapped around the Mg^2+^ ion-bound and -unbound toxins, respectively. The dimeric structure of the *N*-terminal β1-α1-α2 of the antitoxin can be annotated as a RHH domain family, which is commonly found in transcription factors that bind to DNA in a sequence-specific manner. A structural homology search using the program DALI showed that Rv0300 is structurally the closest to the antitoxin FitA from *Neisseria gonorrhoeae* (*N. gonorrhoeae*). The overall octameric structure of the Rv0300–Rv0301 complex is analogous to those of the FitAB complex from *N. gonorrhoeae* [162], the VapBC complex from *Shigella flexneri* (*S. flexneri*) [164], and the VapBC2 complex from *Rickettsia felis* (*R. felis*) [163]. The structure of the toxin shows that its active site is coordinated with one Mg^2+^ ion, suggesting that the catalysis requires at least a single divalent metal ion. In *N. gonorrhoeae*, there are the known structures of a heterotetramer of the FitAB complex and a relatively loose octamer of the FitAB complex bound to a 36-bp DNA fragment from the fitAB promoter (Figure 5A) [162]. The higher-order structure of the DNA-bound form explains why the TA complex enhances the binding affinity of the antitoxin for the DNA. The FitA antitoxin contains the *N*-terminal ribbon-helix-helix (RHH) motif responsible for sequence-dependent DNA binding, and the *C*-terminal toxin-binding region. The FitB toxin shows a high degree of sequential and structural similarity to well-known PIN domains. The conserved residues of the toxin are Asp5, Glu42, Asp104, and Asp122 that are clustered to form an acidic pocket (Figure 5A). The structure of the VapBC complex from *R. felis* was determined in a complex form with a 27-bp double-stranded DNA molecule derived from the promoter was determined [163]. Four toxins and four antitoxins form an octameric structure with 4:4 stoichiometry upon DNA binding (Figure 5B), as in the case of FitAB. However, in the absence of DNA, four toxins and two antitoxins form a hexameric structure with 4:2 stoichiometry. A single antitoxin is sufficient to simultaneously block two putative active sites of homodimeric toxins, as is also found in the MazEF complex [122]. These findings show how the binding stoichiometry can control the balance between the neutralization of toxicity and the transcriptional regulation of the TA operon. Similar to the FitB toxin, the VapC toxin forms a homodimer that contains a PIN-domain. The *N*-terminal DNA-binding domain of the VapB antitoxin forms a swapped-hairpin β-barrel fold that is characteristic of the AbrB/MazE family. This is a good example of the interplay between toxin and antitoxin families. The VapC toxin from *S. flexneri* has a typical PIN domain structure that contains five-stranded β-sheet surrounded by seven α-helices [164]. The VapB antitoxin has the *N*-terminal DNA-binding domain of SpoVT/AbrB type that consists of four-stranded β-sheet, and an extended *C*-terminal tail that wraps around the toxin. These molecules are assembled into a compact heterooctamer in a stoichiometry of 4:4 (VapB_4_C_4_) (Figure 5C). The higher-order structure is also present in solution state, indicative of the strong TA interaction, which is demonstrated by size-exclusion chromatography and chemical cross-linking experiments. The VapBC complex specifically binds to the promoter region including the −10 Pribnow box and the −35 sequences.

### 4.4. YefM–YoeB

**Figure 6:**
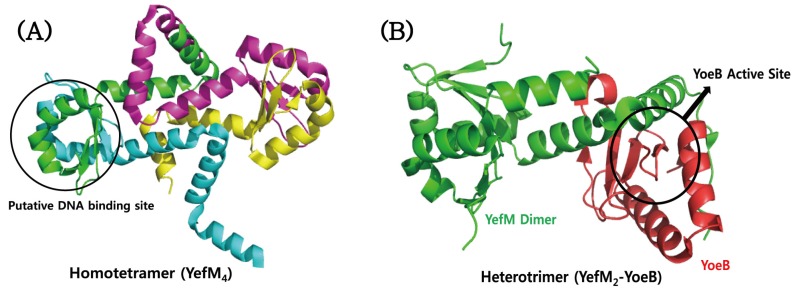
Structures of (**A**) *M. tuberculosis* YefM and (**B**) *E. coli* YefM–YoeB complex. The putative *N*-terminal DNA binding region of *M. tuberculosis* YefM and the active site of *E. coli* YoeB are indicated.

Crystallographic studies demonstrated that the YefM antitoxin of the YefM–YoeB system from *M. tuberculosis* shows a well-defined structure with different conformations of the *C*-terminal part, but not the intrinsically unfolded structure that is commonly adopted by other many antitoxins (Figure 6A) [177]. Multiple-sequence alignments suggest that the polar residues Ser4, Ser6, Arg8, Asp22, Thr29, and Ser39 of the YefM antitoxin are conserved, which might be necessary for DNA binding. On the basis of this conservation, the putative DNA binding site could be mapped onto the YefM structure (Figure 6A). However, the *C*-terminal region of YefM that recognizes its cognate toxin is evolutionarily less conserved. In spite of this, the antitoxin was experimentally proved to neutralize different cognate toxin YoeB from *E. coli*. The structure of *M. tuberculosis* YefM alone was determined as a homotetramer (Figure 6A), but the structure of *E. coli* YefM was determined in a complex form with YoeB, which showed a heterotrimer with a YefM:YoeB stoichiometry of 2:1 (Figure 6B). The structures of two YefM antitoxins from *M. tuberculosis* and *E. coli* are well overlaid with an RMSD value of 1.08 Å for 93 Cα atoms. They show structural differences in the central helices and the distal C-terminal region. It is possible that the conformational change of *E. coli* YefM is induced by its complex formation with the cognate YoeB. Cell viability experiments combined with site-directed mutagenesis demonstrated that four residues of *E. coli* YoeB, Glu46, Arg65, His83, and Tyr84, are required for its RNase activity and cell toxicity [124]. In consistent with this, the three *C*-terminal amino acids of *E. coli* YoeB, Tyr82, His83, and Tyr84, form a canonical fold like a microbial RNase active site. Interestingly, some hydrophobic residues of *M. tuberculosis* YefM for the formation of the tetramer correspond to the residues of *E. coli* YefM that participate in the complex formation with YoeB.

### 4.5. *ε*–*ζ*

**Figure 7:**
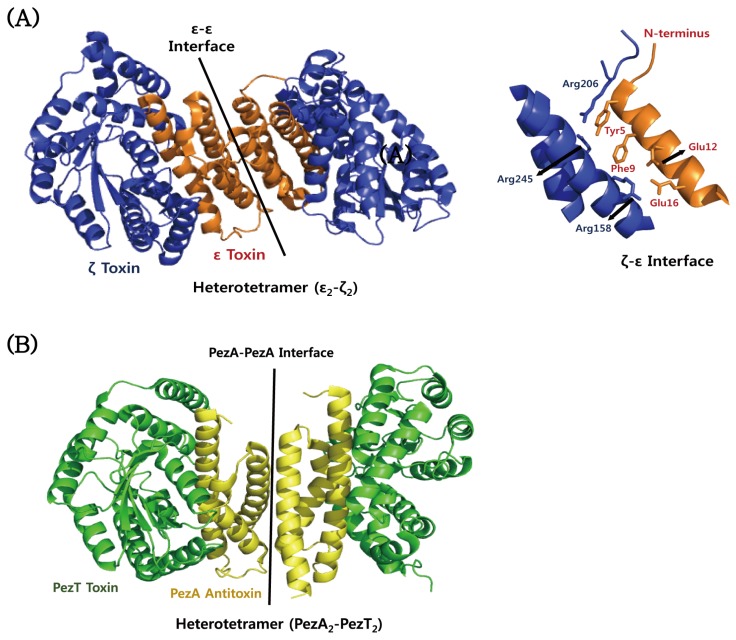
Structural comparison of ε–ζ and PezAT proteins from *S. pyogenes* and *S. pneumonia*. (**A**) Structure and binding interface of the ε–ζ complex from *S. pyogenes*. The residues involved in the ε–ζ interaction are indicated on the right side. (**B**) Structure of the PezAT complex from *S. pneumoniae*.

The crystal structure of the ε–ζ complex from *S. pyogenes* shows a heterotetramer (ε_2_–ζ_2_) in which the antitoxin ε is folded into a three-helices bundle and that the toxin ζ is characteristic of α/β structure with the central twisted β-sheet flanked by α-helix (Figure 7A) [68]. The toxin ζ is structurally similar to a phosphotransferase. Consistently, mutagenesis studies showed that the toxin ζ behaves as a phosphotransferase by using ATP/GTP. The toxicity is inhibited when the *N*-terminal helix of the antitoxin ε occludes the ATP/GTP-binding site of the toxin ζ. The phosphorylated product inhibits MurA [67], a protein that is essential for the initial step of peptidoglycan synthesis in bacteria. Therefore, cell membrane integrity is damaged, resulting in an autolytic phenotype. PezT and ζ toxins share 42% sequence identity and are structurally highly similar [140]. In addition, the *C*-terminal region of PezA shares sequential and structural homology to the ε antitoxin, although the *N*-terminal helix-turn-helix (HTH) motif of PezA functions as transcription repressor similarly to the ω protein. Similar to the ε–ζ complex, the PezAT complex forms a hetero-tetrameric structure in a binding stoichiometry of two PezA and two PezT (Figure 7B). PezA sterically blocks the nucleotide binding site of PezT [140]. The tetramer recognizes a palindrome sequence that overlaps the promoter. Mutagenesis experiments demonstrated that the toxicity of PezT requires a phosphoryl transferase active site and an ATP/GTP-binding site.

### 4.6. Atypical TA Modules

**Figure 8:**
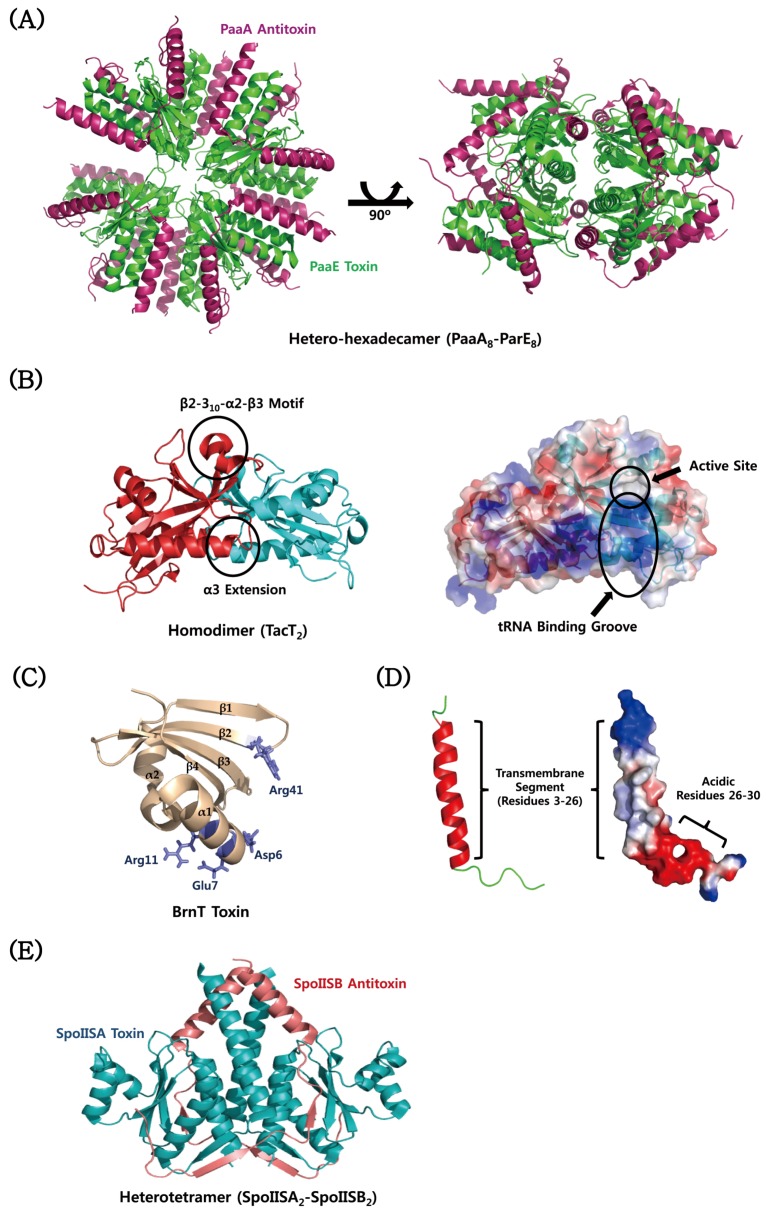
Structures of atypical TA proteins from *E. coli* O157, *S. typhimurium*, *B. abortus*, *E. faecalis*, and *B. subtilis*. (**A**) Structure of the PaaA–ParE complex from *E. coli* O157; (**B**) structure of the TacT toxin from *S. typhimurium*. The tRNA binding and active sites are mapped onto the electrostatic surface model of TacT. (**C**) Structure of the BrnT toxin from *B. abortus*. The residues related to a ribonuclease activity are indicated. (**D**) Structure of the Fst toxin from *E. faecalis*. Transmembrane segment and acidic residues on the electrostatic surface model of Fst are indicated. (**E**) Structure of the SpoIISA–SpoIISB complex.

The EcPaaA2–EcParE2 complex from *E. coli* O157 forms a unique hetero-hexadecamer both in the crystal and in solution [166] (Figure 8A). EcParE2 is structurally most similar to the RelE/ParE toxin family, which folds as two *N*-terminal α-helices followed by a four-stranded antiparallel β-sheet. However, EcParE2 shows a low sequence identity with well-known *E. coli* RelE and YoeB. EcParE2 does not bind to DNA gyrase and also not inhibits protein synthesis, suggesting that the RelE/ParE family has a third, distinct, yet unidentified function. A combination of small-angle X-ray scattering (SAXS) and NMR studies described the conformational ensemble of the EcPaaA2 antitoxin [165], which is an atypical member of the RelB antitoxin family. In solution, the ensemble exhibits an anisotropy in its coverage of the available conformational space compared to initially selected structures. The EcPaaA2 antitoxin is highly flexible and adopts two α-helices connected by a flexible linker. TacT toxin from *S. typhimurium* is a unique type of acetyltransferases in that it adopts an extension of α3 helix and a bending of β2 and β3 strands with an insertion of short helices between them, compared to other acetyltransferases (Figure 8B). This protein forms a homodimer with an extensive dimeric interface and a positively charged groove for tRNA binding (Figure 8B). The structural model suggested that TacT-mediated acetylation of primary amine group of aminoacyl-tRNA is unable to form a peptide bond with the carboxyl group of the polypeptide chain located in the P site of ribosome. A novel type II TA system from *B. abortus* was identified as the BrnAT system [172]; it consists of the toxin BrnT and the antitoxin BrnA, which are co-expressed and form a 2:2 tetrameric complex. As shown in typical type II TA systems, the tetrameric BrnAT complex binds to its own promoter and downregulates its expression. BrnT has a central scaffold that is composed of a four-stranded antiparallel β-sheet with a β2–β3–β4–β5 topology, in which a hydrophobic face binds to two short α-helices (Figure 8C) [172]. The BrnT toxin shows a similar secondary topology to the RelE toxin family, but overall tertiary structural similarity between them is low. Despite the structural differences between BrnT and other RelE family members, they are functionally conserved as ribonucleases that inhibit protein synthesis in vivo. The most conserved residues of the BrnT toxin are situated in the solvent-exposed surfaces of the β-scaffold and its interacting α-helix. A combination of cell toxicity assay and site-directed mutagenesis revealed that eight of the conserved residues are involved in cell toxicity and that five (Asp6, Glu7, Lys9, Arg41, and Glu78) of these eight residues are important for a ribonuclease activity [172]. These residues, except for Glu78 (not shown in the structure), are colour-mapped onto the BrnT structure in Figure 8C. A combined approach of NMR spectroscopy and molecular dynamics simulation revealed that the Fst toxin from *E. faecalis* has the transmembrane segment spanning residues Asp3–Asp26, which form a stable helical structure and are completely immersed in the membrane (Figure 8D) [145]. An intrinsically disordered stretch near the *C*-terminus, including acidic residues (Asp26–Glu27–Glu28–Asp29–Asp30), is located outside the membrane (Figure 8D). The protruding unstructured *C*-terminal region and transmembrane orientation of the Fst toxin is distinct from those of common antimicrobial peptides. This suggests that they have different mechanisms of action, although both perturb membrane integrity and topology. SpoIISA TA system from *B. subtilis* has a higher molecular weight, with 248 amino acid residues, compared to other TA system toxins. This protein is predicted to have transmembrane and cytoplasmic domains. The full-length SpoIISA protein is required for cell toxicity, which is counteracted by a small SpoIISB antitoxin that is a basic hydrophilic protein with 56 amino acid residues. The two proteins have no significant sequence similarity to proteins with known structure or function. The complex structure of the cytoplasmic domain of SpoIISA (residues 80–248) with SpoIISB was determined [178]. The cytosolic domain forms the compact globular structure that consists of five α-helices and five β-strands (Figure 8E). A four-stranded anti-parallel β-sheet with a β2–β5–β4–β3 topology forms the core structure, whose opposite sides are packed by helices 3 and 4 and helices 2 and 5, respectively. In contrast to the SpoIISA toxin, the SpoIISB antitoxin adopts a highly extended conformation. The monomeric antitoxin has an extensive interaction with both subunits of the dimeric toxin (Figure 8E).

## 5. Exploitation of TA Systems for the Development of Novel Antibiotic Drugs

**Figure 9:**
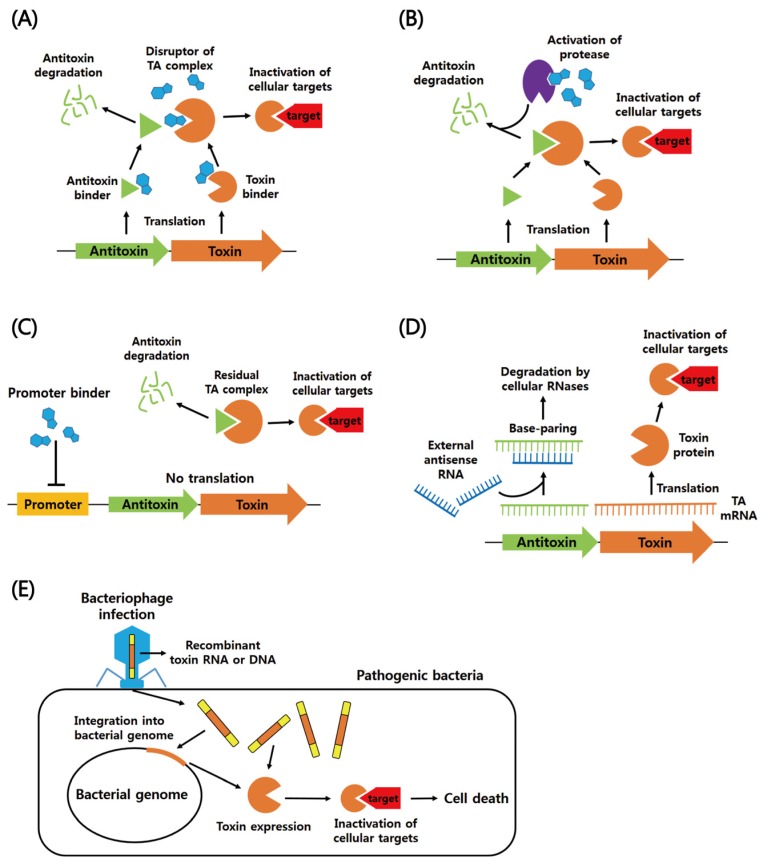
Schematic antibacterial strategies using TA systems. (**A**) Disruptors of TA complex. Direct activation of the toxin is achieved by disruption of the preformed TA complex or prevention of complex formation. The latter can be achieved by either an antitoxin binder or a toxin binder. (**B**) Activation of cellular proteases. A molecule activates cellular proteases, which promotes the degradation of the labile antitoxin protein from the TA complex. (**C**) Inhibition of TA gene transcription. A molecule binds to the promoter of TA operon, inhibiting its transcription. The antitoxin is not replenished, and the remaining antitoxin is degraded. Free toxin acts on cellular targets. (**D**) Inhibition of antitoxin translation. Antisense RNA forms complementary base-paring with antitoxin mRNA, which inhibits antitoxin translation. Free toxin inactivates cellular targets. (**E**) Bacteriophage infection. Recombinant toxin RNA or DNA enter pathogens via bacteriophage infection. The nucleic acids integrate into the bacterial genome in a lysogenic cycle. The recombinant toxins are produced and cause cell death.

Infectious diseases have been considered one of the leading causes of human mortality worldwide. TA systems have recently received much attention as one of the most promising antibacterial targets [90,182,183,184,185] because they are widely distributed in almost all of the most important bacterial pathogens but not in eukaryotic cells. One of the powerful antibacterial strategies is an artificial activation of the toxin, which could be accomplished by using small compounds or peptides as inhibitors of the protein (toxin)-protein (antitoxin) interactions (Figure 9A). However, this field is in the early stages; there are still no drugs designed to target TA interfaces. The identification of drug candidates can be assisted by computational drug design such as molecular docking and virtual screening [186]. Another antibiotic strategy that can be envisioned is to accelerate degradation of the bound antitoxin by Lon or Clp proteases, resulting in a release of the toxin and subsequent cell death (Figure 9B). This approach utilizes the mechanism in which the overproduction of the protease indirectly vitalizes the toxin. In fact, overexpression of Lon protease specifically activates the YoeB toxin from its non-toxic complex with the YefM antitoxin, leading to global translation inhibitions and hence cell death [187]. Antibiotic molecules that bind and activate ClpP protease were identified in other systems [188,189]. It is conceivable that drug candidates could bind to the promoter DNA of a TA operon and repress its transcription, hindering the continuous supply of the short-lived antitoxin within the cell, which eventually causes cell lethality from the free toxin (Figure 9C). To achieve this, biomolecules can be designed to enhance promoter binding and avoid the formation of a complex with the toxin on the basis of the structure of DNA-binding motif of the antitoxin. The three strategies mentioned above utilize type II TA systems, which are found in a large number of bacteria but not in eukaryotes. To inhibit the synthesis of the antitoxin, we could use antisense RNA that binds to the antitoxin coding mRNA without affecting the toxin translation (Figure 9D). This approach could be applied to all types of TA systems. The toxin molecule itself could be an antibiotic drug candidate, but the therapeutic obstacles are poor oral availability and high costs of bioprocessing as shown in many biomolecular drugs. In addition, the toxins have to be effectively delivered to habitats of pathogenic bacteria in the patient’s body without deleterious effects on the human cells or normal commensal organisms. The type I toxin PepA1 from *S. aureus* is a 30-residue hydrophobic peptide that can cause membrane lysis of both bacterial cells and human erythrocytes [190,191]. This toxin was sequentially and chemically optimized to enhance the antibacterial activity and reduce the hemolytic side effect [191]. The mechanism of action of PepA1 derivatives can be explained by their structural property such as linear, helical, and cyclic conformations [191]. A more sophisticated approach would make use of recombinant bacteriophages [192], which deliver the desired toxin gene into pathogenic bacteria. The highly expressed toxin will kill the pathogens (Figure 9E). A variety of toxin genes could be initially acquired from many known TA operons. However, the use of the bacteriophages has been restricted due to their infections for specific organisms.

The most commonly used strategy for the artificial activation of toxins is to use the structural and biochemical information of type II TA proteins to design peptides that inhibit the TA interactions. For example, peptide inhibitors of the *B. anthracis* PemIK interaction were designed to mimic the *C*-terminal toxin-binding region of the antitoxin [193]. The toxin PemK cleaves single-stranded RNAs, and toxicity is inhibited by the antitoxin PemI. Deletion mutant studies showed that the *C*-terminal fragment of PemI is responsible for PemK binding [194]. The two peptides (LLFQHLTE and RRGYIEMG) block the PemIK interaction and inhibit in vitro ribonuclease activity of the PemK toxin, whereas peptides derived from the *N*-terminal fragment of PemI do not influence the TA interaction [193]. A rationally designed octapeptide (SKIGAWAS) is predicted to form an α-helix and to occupy the TA binding interface [194]. Consistent with this prediction, this peptide has been shown to inhibit TA complex formation but also decrease the ribonuclease activity of the toxin. However, in order to be candidates of potent antibiotics, a molecule has to not only disrupt the TA interactions but also fully recover the intrinsic activity of the toxin. The latter might be achieved unless a molecule affects the catalytic site of the toxin. An extensive screening of peptides that disrupt the interaction between the antitoxin ε and the toxin ζ from *S. pyogenes* plasmid was carried out using bioluminescence resonance energy transfer (BRET) signals between Luc–ε and ζ–GFP fusion proteins [186]. A considerable number of peptide libraries including over 4.95 × 10^7^ 6-residue peptides, 2.74 × 10^4^ 14-residue β-sheet peptides, and 2.74 × 10^4^ 17-residue α-helix peptides were tested for their abilities to prevent TA interactions. Among them, only a few members of the 17-residue library were observed to decrease the BRET signal, indicating that they are able to disrupt the TA interaction. On the basis of the crystal structure of the VapBC complex (Rv0623–Rv0624), three peptides that mimic the TA interface were designed [176]: an 8-residue peptide I from the α1-helical region of Rv0623 (residues 52–59), a 17-residue peptide II from the α2-helical region of Rv0624 (residues 14–30), and a nine-residue peptide III from the α4-helical region of Rv0624 (residues 48–56). How much they increased the ribonuclease activity of the VapC toxin from the non-toxic VapBC complex was evaluated. The recovery of the activity could be explained by the peptide that liberates the toxin from the TA complex. As a result, peptides II and III increased the ribonuclease activity of the VapC toxin more than peptide I. This is an example of the toxin-mimicking peptide that inhibits the TA interaction more strongly than the antitoxin-mimicking peptide.

There are critical considerations for the design of antibiotic drugs using TA systems from pathogenic bacteria. Drug candidates, such as peptides or small molecules, should have strong binding affinity to the interface of the TA complex, enough to fully disrupt a stable pre-formed complex, because most known TA complexes are formed with a strong binding affinity. The binding commonly involves extensive electrostatic and hydrophobic interactions; the antitoxin wraps around the toxin. The large interface of the TA complex is difficult to bind strongly to small molecules. This obstacle has been frequently encountered in the development of inhibitors of protein-protein interaction (PPI). However, there are precedents that the binding interface of the protein-protein complex has a long and shallow pocket that is more easily accessible to small molecules [195]. Other issues are related to the limited information on a variety of TA interactions. The sequence and structure of antitoxins are highly divergent, although their cognate toxins show considerable structural similarity to other toxins of the same family. To inhibit the TA interaction, one could design a molecule that binds to the *C*-terminal toxin-binding site of the antitoxin. However, this binding site is a challenging target because it commonly adopts an intrinsically disordered structure. Furthermore, the artificial activation of the toxin has to be effective in cell death when TA operons are highly activated under stressful conditions such as nutritional starvation, antibiotic treatments, and oxidative stresses. There is concern that artificial activation of toxins to a moderate level could induce the formation of persister or dormant cells that contribute to chronic infection. In this regard, another strategy can be envisaged that resuscitates the dormant cell, rendering it susceptible to antibiotic drugs. It remains to systemically validate the effectiveness of diverse antibiotic strategies utilizing TA systems of pathogens.

## Figures and Tables

**Table 1:** Actions of well-characterized toxins in TA systems.

Type	TA Module	Protein Toxin	Antitoxin (Molecular Species)	Toxin Activity	Inhibited Cellular Process	Ref.
I	*istR-tisB*	TisB	*istR* (RNA)	Depolarizes Cell Membrane	ATP Synthesis	[41,42]
I	*sok-hok*	Hok	*sok* (RNA)	Depolarizes Cell Membrane	ATP Synthesis	[43,44]
I	*sibC-ibsC*	IbsC	*sibC* (RNA)	Depolarizes Cell Membrane	ATP Synthesis	[45,46]
I	*ratA-txpA*	TxpA	*ratA* (RNA)	Lyses Cell Membrane	Not Applicable	[47,48]
I	*fst–RNAI–RNAII*	Fst	RNAII (RNA)	Damages Cell Membrane	Cell Division	[49,50]
I	*symRE*	SymE	*symR* (RNA)	mRNA Cleavage	Translation	[51]
II	*mazEF*	MazF	MazE (protein)	mRNA Cleavage	Translation	[52,53]
II	*kis-kid*	Kid	Kis (protein)	mRNA Cleavage	Translation	[54]
II	*higBA*	HigA	HigB (protein)	mRNA Cleavage	Translation	[55,56]
II	*relBE*	RelE	RelB (protein)	mRNA Cleavage	Translation	[57]
II	*vapBC*	VapC	VapB (protein)	RNA Cleavage	Translation	[58,59,60]
II	*phd-doc*	Doc	Phd (protein)	Phosphorylation of Translation Elongation Factor EF-Tu	Translation	[61,62]
II	*hipBA*	HipA	HipB (protein)	Phosphorylation of Glutamyl tRNA Synthetase	Translation	[63,64]
II	*ccdAB*	CcdB	CcdA (protein)	Poison of DNA gyrase	Transcription and Replication	[65,66]
II	*parDE*	ParE	ParD (protein)	Poison of DNA gyrase	Transcription and Replication	[65,66]
II	ω-ε-ζ	ζ	ε (protein)	Phosphorylation of UDP-*N*-acetylglucosamine	Peptidoglycan Synthesis	[67,68]
III	*toxIN*	ToxN	*toxI* (RNA)	RNA Cleavage	Translation	[69]
IV	*yeeUV*	YeeV	YeeU (protein)	Interacts with Cytoskeleton Proteins FtsZ and MreB	Cell Division	[70]
IV	*cptBA*	CptA	CptB (protein)	Interacts with Cytoskeleton Proteins FtsZ and MreB	Cell Division	[71]
V	*ghoST*	GhoT	GhoS (protein)	Damages Cell Membrane	Not Applicable	[72]
VI	*socAB*	SocB	SocA (protein)	Binds to the β sliding clamp DnaN	Replication	[73]

**Table 2:** Structures of TA proteins from pathogenic bacteria described in this review.

Pathogenic Bacteria	TA Protein	Oligomeric State (Stoichiometry)	Pfam Annotation/Accession ID	PDB ID/Deposit Date	Ref.
*Streptococcus pyogenes*	ε-ζ Complex	Hetero-tetramer (A2B2)	ε: Bacterial Epsilon Antitoxin Domain/PF08998	1GVN/2002-02-19	[68]
			ζ: Zeta Toxin Family/PF06414		
*Streptococcus pyogenes*	ε-ζ Bound to the Substrate	Hetero-tetramer (A2B2)	ε: Bacterial Epsilon Antitoxin Domain/PF08998	3Q8X/2011-01-07	[67]
			ζ: Zeta Toxin Family/PF06414		
*Streptococcus pneumoniae*	PezAT Complex	Hetero-tetramer (A2B2)	PezA: 3-layer (αβα) Sandwich Architecture and Rossmann Fold Topology*	2P5T/2007-03-16	[140]
			PezT: Zeta Toxin Family/PF06414		
*Enterococcus faecalis*	Fst Toxin	Monomer (A)	Toxin Fst Domain/PF13955	2KV5/2010-03-08	[145]
*Staphylococcus aureus*	MazF Toxin	Homo-dimer (A2)	PemK-like Protein Family/PF02452	4MZM/2013-09-30	[161]
*Neisseria gonorrhoeae*	FitB Toxin	Homo-dimer (A2)	FitB: PIN Domain/PF01850	2H1C/2006-05-16	[162]
*Neisseria gonorrhoeae*	FitAB Bound to DNA	Hetero-octamer (A4B4)	FitB: PIN Domain/PF01850	2H1O/2006-05-16	[162]
			FitA: 3-layer (αβα) Sandwich Architecture and Rossmann Fold Topology *		
*Rickettsia felis*	VapBC2 Bound to DNA	Hetero-octamer (A4B4)	VapB2: Antitoxin MazE Domain/PF04014	3ZVK/2011-07-25	[163]
			VapC2: PIN Domain/PF01850		
*Shigella flexneri*	YeeU Antitoxin	Monomer (A)	YagB/YeeU/YfjZ Family/PF06154	2INW/2006-10-09	[125]
*Shigella flexneri*	VapBC Complex	Hetero-octamer (A4B4)	VapC: PIN Domain/PF01850	3TND/2011-09-01	[164]
			VapB: Antitoxin MazE Domain/PF04014		
*Escherichia coli* O157	PaaA2 Antitoxin	Monomer (A)	Not Applicable	3ZBE/2012-11-08	[165]
*Escherichia coli* O157	PaaA2-ParE2 Complex	Hetero-heptadecamer (A8B8)	PaaA2: Not Applicable	5CW7/2015-07-27	[166]
			ParE2: Plasmid Stabilization System Protein/PF05016		
*Escherichia coli* K12	MazEF Complex	Hetero-hexamer (A4B2)	MazE: Antidote-toxin Recognition MazE/PF04014	1UB4/2003-03-28	[122]
			MazF: PemK-like Protein/PF02452		
*Escherichia coli* K12	MazF-Substrate Complex	Homo-dimer (A2)	MazF: PemK-like Protein/PF02452	5CR2/2015-07-22	[167]
			Substrate Sequence: d(AUACAUA)		
*Escherichia coli* K12	CcdB Toxin	Homo-dimer	CcdB Protein Domain/PF01845	1VUB/1998-04-17	[168]
*Escherichia coli* K12	CcdB Toxin Bound to Gyrase	Hetero-tetramer (A2B2)	CcdB Protein Domain/PF01845	1X75/2004-08-13	[169]
*Escherichia coli* K12	YefM-YoeB Complex	Hetero-trimer (A2B)	YefM: Antitoxin Phd_YefM Domain/PF02604	2A6Q/2005-07-04	[124]
			YoeB: Plasmid encoded Toxin Txe Family		
*Vibrio fischeri*	CcdB Toxin	Homo-dimer (A2)	CcdB Protein Domain/PF01845	3JSC/2009-09-10	[170]
*Vibrio fischeri*	CcdB Toxin Bound to Gyrase	Hetero-tetramer (A2B2)	CcdB Protein Domain/PF01845	4ELZ/2012-04-11	[171]
*Brucella abortus*	BrnT Toxin	Homo-tetramer (A4)	Protein of Unknown Function/PF04365	3U97/2011-10-18	[172]
*Salmonella typhimurium*	TacT Toxin	Homo-dimer (A2)	Acetyltransferase (GNAT) Domain/PF13673	5FVJ/2016-02-08	[159]
*Mycobacterium tuberculosis*	VapBC5 Complex	Hetero-dimer (AB)	VapB5: Antitoxin Phd_YefM Domain/PF02604	3DBO/2008-06-02	[173]
			VapC5: PIN Domain/PF01850		
*Mycobacterium tuberculosis*	VapBC3 Complex	Hetero-octamer (A4B4)	VapB3: Ribbon-helix-helix Protein, copG Family/PF01402	3H87/2009-04-28	[174]
			VapC3: PIN Domain/PF01850		
*Mycobacterium tuberculosis*	VapBC15 Complex	Hetero-tetramer (A2B2)	VapB15: Uncharacterized, Conserved Protein/PF09957	4CHG/2013-12-02	[175]
			VapC15: PIN Domain/PF01850		
*Mycobacterium tuberculosis*	VapBC30 Complex	Hetero-tetramer (A2B2)	VapB30: Rv0623-like Transcription Factor/PF07704	4XGQ/2015-01-02	[176]
			VapC30: PIN Domain/PF01850		
*Mycobacterium tuberculosis*	YefM Antitoxin	Homo-tetramer (A4)	Antitoxin Phd_YefM Domain/PF02604	3CTO/2008-04-14	[177]
*Bacillus subtilis*	SpoIISA-SpoIISB Complex	Hetero-tetramer (A2B2)	SpoIISB: Antitoxin SpoIISB Family/PF14185	3O6Q/2010-07-29	[178]
			SpoIISA: Toxin SpoIISA Family/PF14171		
*Bacillus subtilis*	MazEF Complex	Hetero-hexamer (A4B2)	MazE: Ribbon-helix-helix Protein, CopG family/PF01402	4ME7/2013-08-25	[179]
			MazF: PemK-like Protein/PF02452		
*Bacillus subtilis*	MazF-Substrate Complex	Homo-dimer (A2)	MazF: PemK-like Protein/PF02452	4MDX/2013-08-23	[179]
			Substrate Sequence: UUdUACAUAA		

* This annotation is according to the CATH protein structure classification.

## Supplementary Materials

The supplementary materials are available online at www.mdpi.com/2072-6651/8/10/305/s1, Table S1: Toxin–Antitoxin pairs of human pathogenic bacteria addressed in the text. The information on TA pairs was collected from the web-based TA database (TADB). The minus sign indicates “Not Applicable”.
