# Supplementary material for: Structure, Biology, and Therapeutic Application of Toxin–Antitoxin Systems in Pathogenic Bacteria
Source: Toxins (Basel). 2016 Oct 22;8(10):305. doi: 10.3390/toxins8100305 (PMC5086665; doi:10.3390/toxins8100305)
Supplement: Supplementary file 1 [file toxins-08-00305-s001.pdf]

Supplementary Materials: Structure, Biology, and Therapeutic Application of Toxin-Antitoxin Systems in Pathogenic Bacteria

Ki-Young Lee and Bong-Jin Lee

Table S1. Toxin–Antitoxin pairs of human pathogenic bacteria addressed in the text. The information on TA pairs was collected from the web-based TA database (TADB) [1]. The minus sign indicates "Not Applicable".

Bacterial Species	Classification (Family/Domain)	Toxin	Antitoxin	Regulator	Experimental Validation
Streptococcus pyogenes M1 GAS	RelBE or parDE/RHH-RelE	SPy_0552	SPy_0550	–	–
Streptococcus pyogenes M1 GAS	mazEF/Xre-MazF	SPy_0938	SPy_0939	–	–
Streptococcus pyogenes M1 GAS	-/-	SPy_1248	SPy_1249	–	–
Streptococcus pyogenes M1 GAS	parDE/RHH-RelE	SPy_1927	SPy_1926	–	–
Streptococcus pyogenes M1 GAS	-/Xre-Bro	SPy_2128	SPy_2127	–	–
Streptococcus pyogenes MGAS10270	RelBE or parDE/RHH-RelE	MGAS10270_Spy0458	MGAS10270_Spy0456	–	–
Streptococcus pyogenes MGAS10270	-/-	MGAS10270_Spy0548	MGAS10270_Spy0549	–	–
Streptococcus pyogenes MGAS10270	hicBA/HicB-HicA	MGAS10270_Spy0569	MGAS10270_Spy0568	–	–
Streptococcus pyogenes MGAS10270	-/Xre-COG2856	MGAS10270_Spy0800	MGAS10270_Spy0801	–	–
Streptococcus pyogenes MGAS10270	-/-	MGAS10270_Spy1346	MGAS10270_Spy1347	–	–
Streptococcus pyogenes MGAS10270	-/Xre-COG2856	MGAS10270_Spy1359	MGAS10270_Spy1358	–	–
Streptococcus pyogenes MGAS10270	-/Xre-COG2856	MGAS10270_Spy1405	MGAS10270_Spy1404	–	–
Streptococcus pyogenes MGAS10270	mazEF, ccd or relBE/RHH-MazF	MGAS10270_Spy1407	MGAS10270_Spy1408	–	–
Streptococcus pyogenes MGAS10270	RelBE or parDE/RHH-RelE	MGAS10270_Spy1710	MGAS10270_Spy1709	–	–

Streptococcus pyogenes MGAS10270	RelBE or parDE/RHH-RelE	MGAS10270_Spy1927	MGAS10270_Spy1928	–	–
Streptococcus pyogenes MGAS10750	RelBE or parDE/RHH-RelE	MGAS10750_Spy0477	MGAS10750_Spy0475	–	–
Streptococcus pyogenes MGAS10750	hicBA/HicB-HicA	MGAS10750_Spy0594	MGAS10750_Spy0593	–	–
Streptococcus pyogenes MGAS10750	-/Xre-COG2856	MGAS10750_Spy0835	MGAS10750_Spy0836	–	–
Streptococcus pyogenes MGAS10750	-/Xre-COG2856	MGAS10750_Spy1713	MGAS10750_Spy1714	–	–
Streptococcus pyogenes MGAS10750	RelBE or parDE/RHH-RelE	MGAS10750_Spy1738	MGAS10750_Spy1737	–	–
Streptococcus pyogenes MGAS10750	-/Xre-Bro	MGAS10750_Spy1901	MGAS10750_Spy1900	–	–
Streptococcus pyogenes MGAS2096	RelBE or parDE/RHH-RelE	MGAS2096_Spy0476	MGAS2096_Spy0475	–	–
Streptococcus pyogenes MGAS2096	-/Xre-COG2856	MGAS2096_Spy1490	MGAS2096_Spy1489	–	–
Streptococcus pyogenes MGAS2096	RelBE or parDE/RHH-RelE	MGAS2096_Spy1666	MGAS2096_Spy1665	–	–
Streptococcus pyogenes MGAS315	parDE/RHH-RelE	SpyM3_0394	SpyM3_0393	–	–
Streptococcus pyogenes MGAS315	-/Xre-COG2856	SpyM3_0683	SpyM3_0684	–	–
Streptococcus pyogenes MGAS315	-/Xre-Bro	SpyM3_0687	SpyM3_0688	–	–
Streptococcus pyogenes MGAS315	-/Xre-COG2856	SpyM3_1264	SpyM3_1263	–	–
Streptococcus pyogenes MGAS315	-/Xre-COG2856	SpyM3_1351	SpyM3_1350	–	–
Streptococcus pyogenes MGAS315	-/Xre-COG2856	SpyM3_1456	SpyM3_1455	–	–
Streptococcus pyogenes MGAS315	parDE/RHH-RelE	SpyM3_1662	SpyM3_1661	–	–
Streptococcus pyogenes MGAS5005	RelBE or parDE/RHH-RelE	M5005_Spy_0457	M5005_Spy_0456	–	–
Streptococcus pyogenes MGAS5005	-/Xre-COG2856	M5005_Spy_1220	M5005_Spy_1219	–	–

Streptococcus pyogenes MGAS5005	-/Xre-COG2856	M5005_Spy_1465	M5005_Spy_1464	–	–
Streptococcus pyogenes MGAS5005	RelBE or parDE/RHH-RelE	M5005_Spy_1641	M5005_Spy_1640	–	–
Streptococcus pyogenes MGAS6180	RelBE or parDE/RHH-RelE	M28_Spy0445	M28_Spy0444	–	–
Streptococcus pyogenes MGAS6180	mazEF/Xre-MazF	M28_Spy1031	M28_Spy1030	–	–
Streptococcus pyogenes MGAS6180	-/Xre-COG2856	M28_Spy1283	M28_Spy1282	–	–
Streptococcus pyogenes MGAS6180	-/Xre-COG2856	M28_Spy1331	M28_Spy1330	–	–
Streptococcus pyogenes MGAS6180	mazEF, ccd or relBE/RHH-MazF	M28_Spy1333	M28_Spy1334	–	–
Streptococcus pyogenes MGAS6180	RelBE or parDE/RHH-RelE	M28_Spy1631	M28_Spy1630	–	–
Streptococcus pyogenes MGAS6180	RelBE or parDE/RHH-RelE	M28_Spy1840	M28_Spy1841	–	–
Streptococcus pyogenes MGAS8232	-/Xre-COG2856	spyM18_0339	spyM18_0340	–	–
Streptococcus pyogenes MGAS8232	-/Xre-Bro	spyM18_0344	spyM18_0345	–	–
Streptococcus pyogenes MGAS8232	parDE/RHH-RelE	spyM18_0617	spyM18_0616	–	–
Streptococcus pyogenes MGAS8232	-/Xre-Bro	spyM18_0723	spyM18_0722	–	–
Streptococcus pyogenes MGAS8232	hicBA/HicB-HicA	spyM18_0747	spyM18_0746	–	–
Streptococcus pyogenes MGAS8232	mazEF/Xre-MazF	spyM18_1307	spyM18_1306	–	–
Streptococcus pyogenes MGAS8232	-/Xre-Bro	spyM18_1802	spyM18_1804	–	–
Streptococcus pyogenes MGAS8232	parDE/RHH-RelE	spyM18_1995	spyM18_1994	–	–
Streptococcus pyogenes MGAS9429	RelBE or parDE/RHH-RelE	MGAS9429_Spy0456	MGAS9429_Spy0455	–	–
Streptococcus pyogenes MGAS9429	hicBA/HicB-HicA	MGAS9429_Spy0565	MGAS9429_Spy0564	–	–

Streptococcus pyogenes MGAS9429	-/Xre-Bro	MGAS9429_Spy0799	MGAS9429_Spy0798	–	–
Streptococcus pyogenes MGAS9429	-/Xre-COG2856	MGAS9429_Spy1467	MGAS9429_Spy1466	–	–
Streptococcus pyogenes MGAS9429	RelBE or parDE/RHH-RelE	MGAS9429_Spy1644	MGAS9429_Spy1643	–	–
Streptococcus pyogenes SSI-1	-/Xre-COG2856	SPs0410	SPs0411	–	–
Streptococcus pyogenes SSI-1	-/Xre-COG2856	SPs0513	SPs0514	–	–
Streptococcus pyogenes SSI-1	-/Xre-COG2856	SPs0599	SPs0600	–	–
Streptococcus pyogenes SSI-1	-/Xre-Bro	SPs1163	SPs1162	–	–
Streptococcus pyogenes SSI-1	-/Xre-COG2856	SPs1169	SPs1168	–	–
Streptococcus pyogenes SSI-1	parDE/RHH-RelE	SPs1460	SPs1461	–	–
Streptococcus pyogenes SSI-1	parDE/RHH-RelE	SPs1660	SPs1659	–	–
Streptococcus pyogenes str. Manfredo	-/Xre-COG2856	SpyM50473	SpyM50474	–	–
Streptococcus pyogenes str. Manfredo	-/Xre-Bro	SpyM50476	SpyM50475	–	–
Streptococcus pyogenes str. Manfredo	-/Xre-COG2856	SpyM50633	SpyM50634	–	–
Streptococcus pyogenes str. Manfredo	-/Xre-COG2856	SpyM51068	SpyM51067	–	–
Streptococcus pyogenes str. Manfredo	hicBA/HicB-HicA	SpyM51292	SpyM51293	–	–
Streptococcus pyogenes str. Manfredo	-/Xre-Bro	SpyM51316	SpyM51317	–	–
Streptococcus pyogenes str. Manfredo	RelBE or parDE/RHH-RelE	SpyM51406	SpyM51407	–	–
Streptococcus pyogenes str. Manfredo	RelBE or parDE/RHH-RelE	SpyM51616	SpyM51615	–	–
Streptococcus pyogenes (plasmid)	omega-epsilon-zeta/-	pSM19035_009	pSM19035_008	pSM19035_007	o

Streptococcus pyogenes (plasmid)	omega-epsilon-zeta/-	pSM19035_012	pSM19035_013	pSM19035_014	o
Streptococcus pyogenes (plasmid)	-/-	pSM19035_013	pSM19035_012	–	–
Streptococcus pneumoniae	pezAT/(antitoxin)	Orf8	Orf9	–	–
Streptococcus pneumoniae CGSP14	RelBE or parDE/RHH-RelE	SPCG_0287	SPCG_0286	–	–
Streptococcus pneumoniae CGSP14	phd-doc/AbrB-Fic	SPCG_0839	SPCG_0838	–	–
Streptococcus pneumoniae CGSP14	RelBE or parDE/RHH-RelE	SPCG_1079	SPCG_1078	–	–
Streptococcus pneumoniae CGSP14	relBE or parDE/PHD-RelE	SPCG_1714	SPCG_1715	–	–
Streptococcus pneumoniae CGSP14	-/Xre-COG2856	SPCG_1910	SPCG_1965	–	–
Streptococcus pneumoniae D39	RelBE or parDE/RHH-RelE	SPD_0256	SPD_0255	–	–
Streptococcus pneumoniae D39	RelBE or parDE/RHH-RelE	SPD_1081	SPD_1082	–	–
Streptococcus pneumoniae D39	relBE or parDE/PHD-RelE	SPD_1550	SPD_1551	–	–
Streptococcus pneumoniae G54	RelBE or parDE/RHH-RelE	SPG_0259	SPG_0258	–	–
Streptococcus pneumoniae G54	phd-doc/AbrB-Fic	SPG_0815	SPG_0814	–	–
Streptococcus pneumoniae G54	RelBE or parDE/RHH-RelE	SPG_1114	SPG_1115	–	–
Streptococcus pneumoniae G54	hicBA/HicB-HicA	SPG_1676	SPG_1675	–	–
Streptococcus pneumoniae G54	-/Xre-COG2856	SPG_1845	SPG_1846	–	–
Streptococcus pneumoniae gamPNI0373	omega-epsilon-zeta/(antitoxin)	HMPREF1038_01132	HMPREF1038_01133	–	–
Streptococcus pneumoniae Hungary19A-6	-/Xre-Bro	SPH_0034	SPH_0035	–	–
Streptococcus pneumoniae Hungary19A-6	-/Xre-COG2856	SPH_0072	SPH_0073	–	–
Streptococcus pneumoniae Hungary19A-6	-/Xre-Bro	SPH_0075	SPH_0074	–	–
Streptococcus pneumoniae Hungary19A-6	RelBE or parDE/RHH-RelE	SPH_0392	SPH_0391	–	–
Streptococcus pneumoniae Hungary19A-6	phd-doc/AbrB-Fic	SPH_0995	SPH_0994	–	–
Streptococcus pneumoniae Hungary19A-6	RelBE or parDE/RHH-RelE	SPH_1337	SPH_1338	–	–
Streptococcus pneumoniae Hungary19A-6	relBE or parDE/PHD-RelE	SPH_1849	SPH_1850	–	–
Streptococcus pneumoniae Hungary19A-6	hicBA/HicB-HicA	SPH_1904	SPH_1903	–	–
Streptococcus pneumoniae Hungary19A-6	-/Xre-COG2856	SPH_2084	SPH_2086	–	–

Streptococcus pneumoniae R6	relBE/RHH-RelE	spr0253	spr0252	–	–
Streptococcus pneumoniae R6	relBE/RHH-RelE	spr1103	spr1104	–	–
Streptococcus pneumoniae R6	relBE/PHD-RelE	spr1585	spr1586	–	–
Streptococcus pneumoniae R6	hicBA/HicB-HicA	spr1613	spr1612	–	–
Streptococcus pneumoniae R6	-/Xre-COG2856	spr1752	spr1753	–	–
Streptococcus pneumoniae TIGR4	relBE/RHH-RelE	SP_0276	SP_0275	–	–
Streptococcus pneumoniae TIGR4	phd-doc/AbrB-Fic	SP_0889	SP_0888	–	–
Streptococcus pneumoniae TIGR4	higBA/Xre-RelE	SP_1143	SP_1144	–	–
Streptococcus pneumoniae TIGR4	relBE/RHH-RelE	SP_1223	SP_1224	–	–
Streptococcus pneumoniae TIGR4	relBE/PHD-RelE	SP_1740	SP_1741	–	–
Streptococcus pneumoniae TIGR4	hicBA/HicB-HicA	SP_1787	SP_1786	–	–
Streptococcus pneumoniae TIGR4	-/Xre-COG2856	SP_1935	SP_1936	–	–
Enterococcus faecalis (plasmid)	-/-	pJHW9-2_006	pJHW9-2_005	–	–
Enterococcus faecalis V583	-/Xre-COG2856	EF0128	EF0129	–	–
Enterococcus faecalis V583	-/HEPN-MNT	EF0137	EF0138	–	–
Enterococcus faecalis V583	-/Xre-COG2856	EF0305	EF0306	–	–
Enterococcus faecalis V583	phd-doc/AbrB-Fic	EF0379	EF0380	–	–
Enterococcus faecalis V583	relBE/RHH-RelE	EF0513	EF0512	–	–
Enterococcus faecalis V583	-/Xre-COG2856	EF1276	EF1277	–	–
Enterococcus faecalis V583	-/Xre-COG2856	EF1421	EF1422	–	–
Enterococcus faecalis V583	-/Xre-COG2856	EF2041	EF2040	–	–
Enterococcus faecalis V583	-/Xre-COG2856	EF2143	EF2142	–	–
Enterococcus faecalis V583	-/HEPN-MNT	EF2535	EF2534	–	–
Enterococcus faecalis V583	-/Xre-COG2856	EF2545	EF2544	–	–
Enterococcus faecalis V583	-/Xre-COG2856	EF2853	EF2852	–	–
Enterococcus faecalis V583	mazEF/AbrB-MazF	EF3262	EF3261	–	–
Enterococcus faecalis V583	RelBE or parDE/RHH-RelE	EFA0066	EFA0065	–	–
Enterococcus faecalis V583	mazEF/AbrB-MazF	EFA0071	EFA0072	–	–
Staphylococcus aureus (plasmid)	omega-epsilon-zeta/-	zeta	epsilon	–	o
Staphylococcus aureus (plasmid)	mazEF/AbrB-MazF	SACH_a18	SACH_a19	–	o
Staphylococcus aureus RF122	-/Xre-Bro	SAB1755c	SAB1756c	–	–
Staphylococcus aureus RF122	mazEF, ccd or relBE/RHH-MazF	SAB1953c	SAB1954c	–	–
Staphylococcus aureus RF122	relBE or parDE/PHD-RelE	SAB2287c	SAB2288c	–	–

Staphylococcus aureus RF122	relBE or parDE/PHD-RelE	SAB2339c	SAB2340c	–	–
Staphylococcus aureus subsp. aureus COL	-/Xre-COG2856	SACOL0889	SACOL0890	–	–
Staphylococcus aureus subsp. *aureus* COL	mazEF/RHH-MazF	SACOL2058	SACOL2059	–	–
Staphylococcus aureus subsp. *aureus* COL	relBE/PHD-RelE	SACOL2404	SACOL2405	–	–
Staphylococcus aureus subsp. *aureus* COL	relBE/PHD-RelE	SACOL2464	SACOL2465	–	–
Staphylococcus aureus subsp. *aureus* MRSA252	-/Xre-COG2856	SAR1557	SAR1556	–	–
Staphylococcus aureus subsp. *aureus* MRSA252	mazEF/RHH-MazF	SAR2156	SAR2157	–	–
Staphylococcus aureus subsp. *aureus* MRSA252	relBE/PHD-RelE	SAR2497	SAR2498	–	–
Staphylococcus aureus subsp. *aureus* MSSA476	-/Xre-COG2856	SAS0896	SAS0897	–	–
Staphylococcus aureus subsp. *aureus* MSSA476	mazEF/RHH-MazF	SAS1973	SAS1974	–	–
Staphylococcus aureus subsp. *aureus* MSSA476	relBE/PHD-RelE	SAS2298	SAS2299	–	–
Staphylococcus aureus subsp. *aureus* MSSA476	relBE/PHD-RelE	SAS2348	SAS2349	–	–
Staphylococcus aureus subsp. *aureus* Mu3	-/Xre-COG2856	SAHV_0846	SAHV_0847	–	–
Staphylococcus aureus subsp. *aureus* Mu3	mazEF, ccd or relBE/RHH-MazF	SAHV_2053	SAHV_2054	–	–
Staphylococcus aureus subsp. *aureus* Mu3	relBE or parDE/PHD-RelE	SAHV_2391	SAHV_2392	–	–
Staphylococcus aureus subsp. *aureus* Mu3	relBE or parDE/PHD-RelE	SAHV_2440	SAHV_2441	–	–
Staphylococcus aureus subsp. *aureus* Mu50	-/Xre-COG2856	SAV0850	SAV0851	–	–
Staphylococcus aureus subsp. *aureus* Mu50	mazEF/RHH-MazF	SAV2068	SAV2069	–	–
Staphylococcus aureus subsp. *aureus* Mu50	relBE/PHD-RelE	SAV2407	SAV2408	–	–
Staphylococcus aureus subsp. *aureus* Mu50	relBE/PHD-RelE	SAV2456	SAV2457	–	–
Staphylococcus aureus subsp. *aureus* MW2	-/Xre-COG2856	MW1437	MW1436	–	–
Staphylococcus aureus subsp. *aureus* MW2	mazEF/RHH-MazF	MW1992	MW1993	–	–
Staphylococcus aureus subsp. *aureus* MW2	relBE/PHD-RelE	MW2329	MW2330	–	–
Staphylococcus aureus subsp. *aureus* MW2	relBE/PHD-RelE	MW2380	MW2381	–	–
Staphylococcus aureus subsp. *aureus* N315	mazEF/RHH-MazF	SA1873	SAS067	–	–
Staphylococcus aureus subsp. *aureus* N315	relBE/PHD-RelE	SA2195	SA2196	–	–
Staphylococcus aureus subsp. *aureus* N315	relBE/PHD-RelE	SA2245	SA2246	–	–
Staphylococcus aureus subsp. *aureus* NCTC 8325	mazEF, ccd or relBE/RHH-MazF	SAOUHSC_02 303	SAOUHSC_02 304	–	–

Staphylococcus aureus subsp. *aureus* NCTC 8325	relBE or parDE/PHD-RelE	SAOUHSC_02691	SAOUHSC_02692	–	–
Staphylococcus aureus subsp. *aureus* NCTC 8325	relBE or parDE/PHD-RelE	SAOUHSC_02756	SAOUHSC_02757	–	–
Staphylococcus aureus subsp. *aureus* str. Newman	-/Xre-COG2856	NWMN_0265	NWMN_0266	–	–
Staphylococcus aureus subsp. *aureus* USA300 TCH1516	-/Xre-COG2856	USA300HOU_0853	USA300HOU_0854	–	–
Staphylococcus aureus subsp. *aureus* USA300 TCH1516	-/Xre-COG2856	USA300HOU_1487	USA300HOU_1486	–	–
Staphylococcus aureus subsp. *aureus* USA300 TCH1516	mazEF, ccd or relBE/RHH-MazF	USA300HOU_2063	USA300HOU_2064	–	–
Staphylococcus aureus subsp. *aureus* USA300 TCH1516	relBE or parDE/PHD-RelE	USA300HOU_2387	USA300HOU_2388	–	–
Staphylococcus aureus subsp. *aureus* USA300 TCH1516	relBE or parDE/PHD-RelE	USA300HOU_2446	USA300HOU_2447	–	–
Neisseria gonorrhoeae FA 1090	mazEF/AbrB-MazF	NGO0516	NGO0517	–	–
Neisseria gonorrhoeae FA 1090	vapBC/RHH-PIN	NGO0907	NGO0908	–	–
Neisseria gonorrhoeae FA 1090	hicBA/HicB-HicA	NGO1628	NGO1627	–	–
Neisseria gonorrhoeae NCCP11945	relBE/Xre-RelE	NGK_0018	NGK_0017	–	–
Neisseria gonorrhoeae NCCP11945	vapBC/RHH-PIN	NGK_0895	NGK_0894	–	–
Neisseria gonorrhoeae NCCP11945	-/RHH-GNAT	NGK_1047	NGK_1046	–	–
Neisseria gonorrhoeae NCCP11945	relBE/AbrB-RelE	NGK_1059	NGK_1058	–	–
Neisseria gonorrhoeae NCCP11945	relBE/Xre-RelE	NGK_1160	NGK_1159	–	–
Neisseria gonorrhoeae NCCP11945	mazEF/AbrB-MazF	NGK_1418	NGK_1417	–	–
Neisseria gonorrhoeae NCCP11945	hicBA/HicB-HicA	NGK_1459	NGK_1460	–	–
Rickettsia felis URRWXCal2	relBE or parDE/PHD-RelE	RF_0016	RF_0015	–	–
Rickettsia felis URRWXCal2	vapBC/AbrB-PIN	RF_0095	RF_0094	–	–
Rickettsia felis URRWXCal2	-/RHH-COG2929	RF_0168	RF_0169	–	–
Rickettsia felis URRWXCal2	hicBA/HicB-HicA	RF_0215	RF_0216	–	–
Rickettsia felis URRWXCal2	vapBC/PHD-PIN	RF_0271	RF_0272	–	–
Rickettsia felis URRWXCal2	-/RHH-COG2929	RF_0312	RF_0311	–	–
Rickettsia felis URRWXCal2	*RelBE or parDE/RHH-RelE*	RF_0334	RF_0335	–	–
Rickettsia felis URRWXCal2	-/HEPN-MNT	RF_0356	RF_0357	–	–
Rickettsia felis URRWXCal2	vapBC/PHD-PIN	RF_0456	RF_0457	–	–
Rickettsia felis URRWXCal2	relBE or parDE/PHD-RelE	RF_0490	RF_0489	–	–

Rickettsia felis URRWXCal2	relBE or parDE/PHD-RelE	RF_0602	RF_0601	–	–
Rickettsia felis URRWXCal2	vapBC/PHD-PIN	RF_0701	RF_0702	–	–
Rickettsia felis URRWXCal2	vapBC/PHD-PIN	RF_0732	RF_0731	–	–
Rickettsia felis URRWXCal2	-/HEPN-MNT	RF_0754	RF_0753	–	–
Rickettsia felis URRWXCal2	relBE or parDE/PHD-RelE	RF_0787	RF_0788	–	–
Rickettsia felis URRWXCal2	RelBE or parDE/RHH-RelE	RF_0898	RF_0899	–	–
Rickettsia felis URRWXCal2	relBE or parDE/PHD-RelE	RF_0911	RF_0910	–	–
Rickettsia felis URRWXCal2	RelBE or parDE/RHH-RelE	RF_0956	RF_0957	–	–
Rickettsia felis URRWXCal2	vapBC/AbrB-PIN	RF_1206	RF_1207	–	–
Rickettsia felis URRWXCal2	RelBE or parDE/RHH-RelE	RF_1272	RF_1273	–	–
Rickettsia felis URRWXCal2	-/HEPN-MNT	RF_1280	RF_1279	–	–
Rickettsia felis URRWXCal2	relBE/Xre-RelE	RF_1286	RF_1287	–	–
Rickettsia felis URRWXCal2	relBE/Xre-RelE	RF_1317	RF_1316	–	–
Rickettsia felis URRWXCal2	mazEF, ccd or relBE/RHH-MazF	RF_1343	RF_1342	–	–
Rickettsia felis URRWXCal2	relBE or parDE/PHD-RelE	RF_1368	RF_1369	–	–
Shigella flexneri 2a str. 2457T	-/RHH-GNAT	S4812	S0373	–	–
Shigella flexneri 2a str. 2457T	relBE/RHH-RelE	S1669	S1670	–	–
Shigella flexneri 2a str. 2457T	relBE/Xre-RelE	S3330	S3329	–	–
Shigella flexneri 2a str. 2457T	-/YhfG-Fic	S4383	S4382	–	–
Shigella flexneri 2a str. 301	-/RHH-GNAT	SF0367	SF0368	–	–
Shigella flexneri 2a str. 301	relBE/RHH-RelE	SF1548	SF1549	–	–
Shigella flexneri 2a str. 301	relBE/-	SF2076	SF2079	–	–
Shigella flexneri 2a str. 301	relBE/Xre-RelE	SF3123	SF3122	–	–
Shigella flexneri 2a str. 301	-/YhfG-Fic	SF3380	SF3381	–	–
Shigella flexneri 2a str. 301	mazEF/AbrB-MazF	SF3434	SF3433	–	–
Shigella flexneri 2b	parD (pem)/-	R100p114	R100p113	–	o
Shigella flexneri 5a str. M90T	vapBC/PHD-PIN	pWR501_0259	pWR501_0258	–	o
Shigella flexneri 5 str. 8401	-/RHH-GNAT	SFV_0397	SFV_0398	–	–
Shigella flexneri 5 str. 8401	relBE/Xre-RelE	SFV_3124	SFV_3123	–	–
Shigella flexneri 5 str. 8401	-/YhfG-Fic	SFV_3367	SFV_3368	–	–
Escherichia coli O157:H7 str. EDL933	ccd/RHH-MazF	Z0057	Z0056	–	o
Escherichia coli O157:H7 str. EDL933	RelBE or parDE/RHH-RelE	Z0284	Z0285	–	–

Escherichia coli O157:H7 str. EDL933	parDE/RHH-RelE	Z0510	Z0509	–	o
Escherichia coli O157:H7 str. EDL933	relBE/Xre-RelE	Z1060	Z1059	–	–
Escherichia coli O157:H7 str. EDL933	higBA/COG5606-RelE	Z3230	Z3231	–	–
Escherichia coli O157:H7 str. EDL933	mazEF/AbrB-MazF	Z4097	Z4098	–	–
Escherichia coli O157:H7 str. EDL933	relBE/Xre-RelE	Z4436	Z4435	–	–
Escherichia coli O157:H7 str. EDL933	prlF-yhaV/AbrB-RelE	Z4482	Z4481	–	–
Escherichia coli O157:H7 str. EDL933	-/YhfG-Fic	Z4722	Z4723	–	–
Escherichia coli O157:H7 str. EDL933	-/RHH-GNAT	Z4777	Z4776	–	–
Escherichia coli O157:H7 str. EDL933	-/RHH-GNAT	Z4832	Z4833	–	–
Escherichia coli O157:H7 str. EDL933	hicBA/HicB-HicA	Z4883	Z4882	–	–
Escherichia coli O157:H7 str. EDL933	relBE/Xre-RelE	Z5150	Z5151	–	–
Escherichia coli O157:H7 str. EDL933	mazEF/AbrB-MazF	Z5836	Z5835	–	–
Escherichia coli O157:H7 str. Sakai	-/-	pOSAK1_02	pOSAK1_03	–	o
Escherichia coli O157:H7 str. Sakai	mazEF, ccd or relBE/RHH-MazF	pO157p29	pO157p28	–	–
Escherichia coli O157:H7 str. Sakai	RelBE or parDE/RHH-RelE	pO157p70	pO157p69	–	–
Escherichia coli O157:H7 str. Sakai	ccd/RHH-MazF	ECs0053	ECs0052	–	–
Escherichia coli O157:H7 str. Sakai	relBE/RHH-RelE	ECs0252	ECs0253	–	–
Escherichia coli O157:H7 str. Sakai	parDE/RHH-RelE	ECs0462	ECs0461	–	–
Escherichia coli O157:H7 str. Sakai	relBE/Xre-RelE	ECs5400	ECs5399	–	–
Escherichia coli O157:H7 str. Sakai	paaR-paaA-parE/RHH-RelE	ECs1067	ECs1068	ECs1069	o
Escherichia coli O157:H7 str. Sakai	paaR-paaA-parE/RHH-RelE	ECs2281	ECs2280	ECs2279	o
Escherichia coli O157:H7 str. Sakai	higBA/COG5606-RelE	ECs2869	ECs2870	–	–
Escherichia coli O157:H7 str. Sakai	mazEF/AbrB-MazF	ECs3642	ECs3643	–	–
Escherichia coli O157:H7 str. Sakai	relBE/Xre-RelE	ECs3965	ECs3964	–	–
Escherichia coli O157:H7 str. Sakai	prlF-yhaV/AbrB-RelE	ECs4008	ECs4007	–	–
Escherichia coli O157:H7 str. Sakai	-/YhfG-Fic	ECs4212	ECs4213	–	–
Escherichia coli O157:H7 str. Sakai	-/RHH-GNAT	ECs4262	ECs4261	–	–

Escherichia coli O157:H7 str. Sakai	-/RHH-GNAT	ECs4307	ECs4308	–	–
Escherichia coli O157:H7 str. Sakai	hicBA/HicB-HicA	ECs4357	ECs4356	–	–
Escherichia coli O157:H7 str. Sakai	relBE/Xre-RelE	ECs4597	ECs4598	–	–
Escherichia coli O157:H7 str. Sakai	mazEF/AbrB-MazF	ECs5203	ECs5202	–	–
Vibrio fischeri ES114	hipBA/Xre-HipA	VF_1470	VF_1469	–	–
Vibrio fischeri MJ11	relBE/Xre-RelE	VFMJ11_1428	VFMJ11_1427	–	–
Vibrio fischeri MJ11	hipBA/Xre-HipA	VFMJ11_1565	VFMJ11_1564	–	–
Vibrio fischeri MJ11	hipBA/Xre-HipA	VFMJ11_2144	VFMJ11_2145	–	–
Vibrio cholerae MJ-1236	mosAT/-	VCD_003702	VCD_003703	–	o
Vibrio cholerae O1 biovar El Tor str. N16961	parDE/PHD-RelE	VCA0311	VCA0312	–	o
Vibrio cholerae O1 biovar El Tor str. N16961	-/RHH-GNAT	VCA0318	VCA0319	–	–
Vibrio cholerae O1 biovar El Tor str. N16961	relBE/RHH-RelE	VCA0323	VCA0324	–	–
Vibrio cholerae O1 biovar El Tor str. N16961	-/RHH-COG2929	VCA0332	VCA0333	–	–
Vibrio cholerae O1 biovar El Tor str. N16961	-/RHH-COG2929(toxin)	VCA0348		–	–
Vibrio cholerae O1 biovar El Tor str. N16961	parDE/-	VCA0359	VCA0360	–	o
Vibrio cholerae O1 biovar El Tor str. N16961	parDE/PHD-RelE	VCA0385	VCA0386	–	o
Vibrio cholerae O1 biovar El Tor str. N16961	higBA/Xre-RelE	VCA0391	VCA0392	–	o
Vibrio cholerae O1 biovar El Tor str. N16961	relBE/PHD-RelE	VCA0423	VCA0422	–	–
Vibrio cholerae O1 biovar El Tor str. N16961	relBE/PHD-RelE	VCA0444	VCA0445	–	–
Vibrio cholerae O1 biovar El Tor str. N16961	higBA/Xre-RelE	VCA0468	VCA0469	–	o
Vibrio cholerae O1 biovar El Tor str. N16961	relBE/PHD-RelE	VCA0478	VCA0477	–	–
Vibrio cholerae O1 biovar El Tor str. N16961	-/RHH-GNAT	VCA0481	VCA0482	–	–
Vibrio cholerae O1 biovar El Tor str. N16961	-/RHH-GNAT(toxin)	VCA0487		–	–
Vibrio cholerae O1 biovar El Tor str. N16961	relBE/PHD-RelE	VCA0489	VCA0488	–	–
Vibrio cholerae O1 biovar El Tor str. N16961	-/PHD-RelE(toxin)	VCA0503		–	–

Vibrio cholerae O395	RelBE or parDE/RHH-RelE	VC0395_0757	VC0395_0756	–	–
Vibrio cholerae O395	-/RHH-GNAT	VC0395_0759	VC0395_0760	–	–
Vibrio cholerae O395	-/RHH-GNAT	VC0395_0765	VC0395_0764	–	–
Vibrio cholerae O395	relBE or parDE/PHD-RelE	VC0395_0794	VC0395_0795	–	–
Vibrio cholerae O395	relBE/Xre-RelE	VC0395_0804	VC0395_0803	–	–
Vibrio cholerae O395	relBE or parDE/PHD-RelE	VC0395_0823	VC0395_0822	–	–
Vibrio cholerae O395	relBE or parDE/PHD-RelE	VC0395_0849	VC0395_0850	–	–
Vibrio cholerae O395	relBE or parDE/PHD-RelE	VC0395_0861	VC0395_0862	–	–
Vibrio cholerae O395	-/RHH-GNAT	VC0395_0916	VC0395_0915	–	–
Vibrio cholerae O395	relBE/Xre-RelE	VC0395_0930	VC0395_0929	–	–
Vibrio cholerae O395	relBE or parDE/PHD-RelE	VC0395_0937	VC0395_0936	–	–
Vibrio cholerae O395	hipBA/Xre-HipA	VC0395_A0340	VC0395_A0339	–	–
Vibrio parahaemolyticus RIMD 2210633	hipBA/Xre-HipA	VP0402	VP0403	–	–
Vibrio parahaemolyticus RIMD 2210633	relBE/PHD-RelE	VP1820	VP1821	–	–
Vibrio parahaemolyticus RIMD 2210633	relBE/RHH-RelE	VP1830	VP1829	–	–
Vibrio parahaemolyticus RIMD 2210633	RelBE or parDE/RHH-RelE	VP1843	VP1842	–	–
Vibrio parahaemolyticus RIMD 2210633	RelBE or parDE/RHH-RelE	VP1843	VP1842	–	o
Vibrio parahaemolyticus RIMD 2210633	parDE/RHH-RelE(antitoxin)	VP1884	VP1885	–	–
Vibrio parahaemolyticus RIMD 2210633	parDE/PHD-RelE	VP1885	VP1886	–	–
Vibrio vulnificus CMCP6	RelBE or parDE/RHH-RelE	VV1_0110	VV1_0111	–	–
Vibrio vulnificus CMCP6	hipBA/Xre-HipA	VV1_0401	VV1_0402	–	–
Vibrio vulnificus CMCP6	hipBA/Xre-HipA	VV1_2047	VV1_2046	–	–
Vibrio vulnificus CMCP6	hipBA/Xre-HipA	VV1_2190	VV1_2191	–	–
Vibrio vulnificus CMCP6	relBE/PHD-RelE	VV1_2410	VV1_2409	–	–
Vibrio vulnificus CMCP6	relBE/PHD-RelE	VV1_2433	VV1_2432	–	–
Vibrio vulnificus CMCP6	parDE/RHH-RelE	VV1_2525	VV1_2526	–	–
Vibrio vulnificus CMCP6	-/RHH-GNAT	VV1_2537	VV1_2538	–	–
Vibrio vulnificus CMCP6	relBE/PHD-RelE	VV1_2547	VV1_2546	–	–
Vibrio vulnificus YJ016	hicBA/HicB-HicA	VVP65	VVP66	–	–
Vibrio vulnificus YJ016	relBE/RHH-RelE	VVA0314	VVA0313	–	–

Brucella abortus bv. 1 str. 9-941	-/Xre-COG2856	BruAb1_0264	BruAb1_0263	–	–
Brucella abortus bv. 1 str. 9-941	RelBE or parDE/RHH-RelE	BruAb1_0430	BruAb1_0431	–	–
Brucella abortus bv. 1 str. 9-941	phd-doc/AbrB-Fic	BruAb1_0579	BruAb1_0580	–	–
Brucella abortus bv. 1 str. 9-941	-/RHH-COG2929	BruAb1_0981	BruAb1_0980	–	–
Brucella abortus S19	-/COG5642-COG5654	BAbS19_II08620	BAbS19_II08610	–	–
Salmonella enterica subsp. *enterica serovar Typhimurium* str. LT2	relBE/PHD-RelE	STM1550	STM1551	–	–
Salmonella enterica subsp. *enterica serovar Typhimurium* str. LT2	-/RHH-GNAT	STM2905	STM2904	–	–
Salmonella enterica subsp. *enterica serovar Typhimurium* str. LT2	RelBE or parDE/RHH-RelE	STM2954.1n	STM2955.S	–	–
Salmonella enterica subsp. *enterica serovar Typhimurium* str. LT2	vapBC/AbrB-PIN	STM3033	STM3034	–	o
Salmonella enterica subsp. *enterica serovar Typhimurium* str. LT2	-/YhfG-Fic	STM3470	STM3471	–	–
Salmonella enterica subsp. *enterica serovar Typhimurium* str. LT2	relBE/RHH-RelE	STM3516	STM3517	–	–
Salmonella enterica subsp. *enterica serovar Typhimurium* str. LT2	phd-doc/PHD-Fic	STM3558	STM3559	–	o
Salmonella enterica subsp. *enterica serovar Typhimurium* str. LT2	-/RHH-GNAT	STM3651	STM3652	–	–
Salmonella enterica subsp. *enterica serovar Typhimurium* str. LT2	relBE/Xre-RelE	STM3777	STM3778	–	–
Salmonella enterica subsp. *enterica serovar Typhimurium* str. LT2	higBA/COG5606-RelE	STM3906	STM3907	–	–
Salmonella enterica subsp. *enterica serovar Typhimurium* str. LT2	relBE/Xre-RelE	STM4031	STM4030.S	–	–
Salmonella enterica subsp. *enterica serovar Typhimurium* str. LT2	relBE/Xre-RelE	STM4031	STM4030.S	–	o
Salmonella enterica subsp. *enterica serovar Typhimurium* str. LT2	relBE/Xre-RelE	STM4032.2N	STM4033	–	o
Salmonella enterica subsp. *enterica serovar Typhimuriu*m str. LT2	relBE/Xre-RelE	STM4032.2N	STM4033	–	–

Salmonella enterica subsp. *enterica serovar Typhimurium* str. LT2	-/RHH-GNAT	STM4318	STM4317	–	–
Salmonella enterica subsp. *enterica serovar Typhimurium* str. LT2	relBE/RHH-RelE	STM4450	STM4449	–	–
Salmonella enterica subsp. *enterica serovar Typhimurium* str. *LT2*	-/RHH-COG2929	STM4528	STM4529	–	–
Salmonella enterica subsp. *enterica serovar Typhimurium* str. LT2	-/RHH-COG2929	STM4528	STM4529	–	o
Salmonella enterica subsp. *enterica serovar Typhi* str. CT18	phd-doc/-	STY1031		–	–
Salmonella enterica subsp. *enterica serovar Typhi* str. CT18	-/RHH-GNAT	STY3027	STY3026	–	–
Salmonella enterica subsp. *enterica serovar Typhi* str. CT18	vapBC/AbrB-PIN	STY3182	STY3183	–	–
Salmonella enterica subsp. *enterica serovar Typhi* str. CT18	higBA/COG5606-RelE	STY3651	STY3650	–	–
Salmonella enterica subsp. *enterica serovar Typhi* str. CT18	vapBC/RHH-PIN	STY3844	STY3845	–	–
Salmonella enterica subsp. *enterica serovar Typhi* str. CT18	relBE/Xre-RelE	STY3847	STY3848	–	–
Salmonella enterica subsp. *enterica serovar Typhi* str. CT18	-/RHH-GNAT	STY4151	STY4150	–	–
Salmonella enterica subsp. *enterica serovar Typhi* str. CT18	-/YhfG-Fic	STY4326	STY4325	–	–
Salmonella enterica subsp. *enterica serovar Typhi* str. CT18	-/RHH-GNAT	STY4518	STY4517	–	–
Salmonella enterica subsp. *enterica serovar Typhi* str. CT18	-/RHH-GNAT	STY4668	STY4667	–	–
Salmonella enterica subsp. *enterica serovar Typhi* str. CT18	relBE/RHH-RelE	STY4789	STY4788	–	–
Salmonella enterica subsp. *enterica serovar Typhi* str. CT18	-/RHH-COG2929	STY4886	STY4887	–	–

Salmonella enterica subsp. *enterica serovar Typhi* str. Ty2	phd-doc/-	t1909	t1910	–	–
Salmonella enterica subsp. *enterica serovar Typhi* str. Ty2	-/RHH-GNAT	t2805	t2804	–	–
Salmonella enterica subsp. *enterica serovar Typhi* str. Ty2	vapBC/AbrB-PIN	t2943	t2944	–	–
Salmonella enterica subsp. *enterica serovar Typhi* str. Ty2	higBA/COG5606-RelE	t3392	t3391	–	–
Salmonella enterica subsp. *enterica serovar Typhi* str. Ty2	vapBC/RHH-PIN	t3587	t3588	–	–
Salmonella enterica subsp. *enterica serovar Typhi* str. Ty2	relBE/Xre-RelE	t3590	t3591	–	–
Salmonella enterica subsp. *enterica serovar Typhi* str. Ty2	-/RHH-GNAT	t3868	t3867	–	–
Salmonella enterica subsp. *enterica serovar Typhi* str. Ty2	-/YhfG-Fic	t4035	t4034	–	–
Salmonella enterica subsp. *enterica serovar Typhi* str. Ty2	-/RHH-GNAT	t4224	t4223	–	–
Salmonella enterica subsp. *enterica serovar Typhi* str. Ty2	-/RHH-GNAT	t4359	t4358	–	–
Salmonella enterica subsp. *enterica serovar Typhi* str. Ty2	relBE/RHH-RelE	t4484	t4483	–	–
Salmonella enterica subsp. *enterica serovar Typhi* str. Ty2	-/RHH-COG2929	t4578	t4579	–	–
Mycobacterium tuberculosis CDC1551	vapBC/RHH-PIN	MT0254	MT0253	–	–
Mycobacterium tuberculosis CDC1551	vapBC/RHH-PIN	MT0289	MT0290	–	–
Mycobacterium tuberculosis CDC1551	vapBC/RHH-PIN	MT0574	MT0575	–	–
Mycobacterium tuberculosis CDC1551	vapBC/PHD-PIN	MT0625	MT0626	–	–
Mycobacterium tuberculosis CDC1551	vapBC/AbrB-PIN	MT0628	MT0629	–	–
Mycobacterium tuberculosis CDC1551	vapBC/RHH-PIN	MT0638	MT0637	–	–
Mycobacterium tuberculosis CDC1551	vapBC/RHH-PIN	MT0646	MT0645.2	–	–
Mycobacterium tuberculosis CDC1551	vapBC/RHH-PIN	MT0652	MT0651	–	–
Mycobacterium tuberculosis CDC1551	vapBC/RHH-PIN	MT0685	MT0686	–	–
Mycobacterium tuberculosis CDC1551	mazEF/RHH-MazF	MT0688	MT0689	–	–

Mycobacterium tuberculosis CDC1551	vapBC/RHH-PIN	MT0690	MT0691	–	–
Mycobacterium tuberculosis CDC1551	vapBC/PHD-PIN	MT0693	MT0692.1	–	–
Mycobacterium tuberculosis CDC1551	vapBC/RHH-PIN	MT0773	MT0772.6	–	–
Mycobacterium tuberculosis CDC1551	-/RHH-GNAT	MT0945	MT0944	–	–
Mycobacterium tuberculosis CDC1551	vapBC/-	MT0988	MT0987	–	–
Mycobacterium tuberculosis CDC1551	mazEF/-	MT1134	MT1135	–	–
Mycobacterium tuberculosis CDC1551	vapBC/RHH-PIN	MT1144	MT1143.1	–	–
Mycobacterium tuberculosis CDC1551	vapBC/RHH-PIN	MT1280	MT1279	–	–
Mycobacterium tuberculosis CDC1551	vapBC/RHH-PIN	MT1441	MT1442	–	–
Mycobacterium tuberculosis CDC1551	mazEF/-	MT1542	MT1541	–	–
Mycobacterium tuberculosis CDC1551	vapBC/RHH-PIN	MT1612	MT1611	–	–
Mycobacterium tuberculosis CDC1551	vapBC/RHH-PIN	MT1761	MT1762	–	–
Mycobacterium tuberculosis CDC1551	vapBC/RHH-PIN	MT1783	MT1782	–	–
Mycobacterium tuberculosis CDC1551	vapBC/RHH-PIN	MT1886	MT1887	–	–
Mycobacterium tuberculosis CDC1551	vapBC/RHH-PIN	MT2003	MT2002	–	–
Mycobacterium tuberculosis CDC1551	relBE/Xre-RelE	MT2004	MT2005	–	–
Mycobacterium tuberculosis CDC1551	parDE/RHH-RelE	MT2008	MT2009	–	–
Mycobacterium tuberculosis CDC1551	vapBC/PHD-PIN	MT2012	MT2013	–	–
Mycobacterium tuberculosis CDC1551	vapBC/RHH-PIN	MT2034	MT2035	–	–
Mycobacterium tuberculosis CDC1551	-/COG5642-COG5654	MT2043	MT2044	–	–
Mycobacterium tuberculosis CDC1551	mazEF/RHH-MazF	MT2046	MT2047	–	–
Mycobacterium tuberculosis CDC1551	vapBC/RHH-PIN	MT2065	MT2064.1	–	–

Mycobacterium tuberculosis CDC1551	-/COG2442-PIN	MT2075	MT2074	–	–
Mycobacterium tuberculosis CDC1551	relBE/Xre-RelE	MT2078	MT2077	–	–
Mycobacterium tuberculosis CDC1551	-/ArsR-COG3832	MT2095	MT2094	–	–
Mycobacterium tuberculosis CDC1551	mazEF, ccd or relBE/RHH-MazF	MT2123	MT2122	–	–
Mycobacterium tuberculosis CDC1551	vapBC/RHH-PIN	MT2163	MT2164	–	–
Mycobacterium tuberculosis CDC1551	parDE/-	MT2200	MT2201	–	–
Mycobacterium tuberculosis CDC1551	vapBC/RHH-PIN	MT2569	MT2568	–	–
Mycobacterium tuberculosis CDC1551	vapBC/RHH-PIN	MT2602	MT2601.2	–	–
Mycobacterium tuberculosis CDC1551	vapBC/RHH-PIN	MT2605	MT2606	–	–
Mycobacterium tuberculosis CDC1551	vapBC/RHH-PIN	MT2622	MT2621	–	–
Mycobacterium tuberculosis CDC1551	vapBC/RHH-PIN	MT2624	MT2623	–	–
Mycobacterium tuberculosis CDC1551	vapBC/RHH-PIN	MT2677	MT2676	–	–
Mycobacterium tuberculosis CDC1551	mazEF/XF1863-MazF	MT2722	MT2721	–	–
Mycobacterium tuberculosis CDC1551	vapBC/RHH-PIN	MT2827	MT2828	–	–
Mycobacterium tuberculosis CDC1551	vapBC/RHH-PIN	MT2829	MT2830	–	–
Mycobacterium tuberculosis CDC1551	vapBC/-	MT2932	MT2931	–	–
Mycobacterium tuberculosis CDC1551	vapBC/PHD-PIN	MT3271	MT3272	–	–
Mycobacterium tuberculosis CDC1551	relBE/Xre-RelE	MT3274	MT3275	–	–
Mycobacterium tuberculosis CDC1551	-/Xre-COG5654	MT3277	MT3276	–	–
Mycobacterium tuberculosis CDC1551	vapBC/RHH-PIN	MT3421	MT3422	–	–
Mycobacterium tuberculosis CDC1551	relBE/PHD-RelE	MT3466	MT3465	–	–
Mycobacterium tuberculosis CDC1551	vapBC/PHD-PIN	MT3492	MT3493	–	–

Mycobacterium tuberculosis CDC1551	vapBC/PHD-PIN	MT3516	MT3515	–	–
Mycobacterium tuberculosis CDC1551	vapBC/RHH-PIN	MT3799	MT3800	–	–
Mycobacterium tuberculosis CDC1551	-/MerR-PIN	MT3856	MT3857	–	–
Mycobacterium tuberculosis F11	vapBC/RHH-PIN	TBFG_10243	TBFG_10242	–	–
Mycobacterium tuberculosis F11	vapBC/RHH-PIN	TBFG_10281	TBFG_10282	–	–
Mycobacterium tuberculosis F11	mazEF, ccd or relBE/RHH-MazF	TBFG_10306	TBFG_10305	–	–
Mycobacterium tuberculosis F11	vapBC/RHH-PIN	TBFG_10593	TBFG_10592	–	–
Mycobacterium tuberculosis F11	vapBC/PHD-PIN	TBFG_10607	TBFG_10608	–	–
Mycobacterium tuberculosis F11	vapBC/AbrB-PIN	TBFG_10610	TBFG_10611	–	–
Mycobacterium tuberculosis F11	vapBC/RHH-PIN	TBFG_10620	TBFG_10619	–	–
Mycobacterium tuberculosis F11	vapBC/RHH-PIN	TBFG_10635	TBFG_10634	–	–
Mycobacterium tuberculosis F11	vapBC/PHD-PIN	TBFG_10638	TBFG_10637	–	–
Mycobacterium tuberculosis F11	vapBC/RHH-PIN	TBFG_10669	TBFG_10670	–	–
Mycobacterium tuberculosis F11	mazEF, ccd or relBE/RHH-MazF	TBFG_10672	TBFG_10673	–	–
Mycobacterium tuberculosis F11	vapBC/RHH-PIN	TBFG_10674	TBFG_10675	–	–
Mycobacterium tuberculosis F11	vapBC/RHH-PIN	TBFG_10763	TBFG_10762	–	–
Mycobacterium tuberculosis F11	-/RHH-GNAT	TBFG_10937	TBFG_10936	–	–
Mycobacterium tuberculosis F11	vapBC/RHH-PIN	TBFG_11136	TBFG_11135	–	–
Mycobacterium tuberculosis F11	vapBC/RHH-PIN	TBFG_11267	TBFG_11266	–	–
Mycobacterium tuberculosis F11	relBE or parDE/PHD-RelE	TBFG_11272	TBFG_11273	–	–
Mycobacterium tuberculosis F11	vapBC/RHH-PIN	TBFG_11426	TBFG_11427	–	–
Mycobacterium tuberculosis F11	vapBC/RHH-PIN	TBFG_11593	TBFG_11592	–	–
Mycobacterium tuberculosis F11	vapBC/RHH-PIN	TBFG_11759	TBFG_11758	–	–
Mycobacterium tuberculosis F11	vapBC/RHH-PIN	TBFG_11982	TBFG_11981	–	–
Mycobacterium tuberculosis F11	relBE/Xre-RelE	TBFG_11983	TBFG_11984	–	–
Mycobacterium tuberculosis F11	RelBE or parDE/RHH-RelE	TBFG_11987	TBFG_11988	–	–
Mycobacterium tuberculosis F11	vapBC/PHD-PIN	TBFG_11990	TBFG_11991	–	–
Mycobacterium tuberculosis F11	-/COG5642-COG5654	TBFG_12020	TBFG_12021	–	–
Mycobacterium tuberculosis F11	vapBC/RHH-PIN	TBFG_12042	TBFG_12041	–	–
Mycobacterium tuberculosis F11	-/COG2442-PIN	TBFG_12054	TBFG_12053	–	–
Mycobacterium tuberculosis F11	relBE/Xre-RelE	TBFG_12057	TBFG_12056	–	–

Mycobacterium tuberculosis F11	-/ArsR-COG3832	TBFG_12072	TBFG_12071	–	–
Mycobacterium tuberculosis F11	vapBC/RHH-PIN	TBFG_12137	TBFG_12138	–	–
Mycobacterium tuberculosis F11	vapBC/RHH-PIN	TBFG_12566	TBFG_12565	–	–
Mycobacterium tuberculosis F11	vapBC/RHH-PIN	TBFG_12568	TBFG_12567	–	–
Mycobacterium tuberculosis F11	vapBC/RHH-PIN	TBFG_12570	TBFG_12571	–	–
Mycobacterium tuberculosis F11	vapBC/RHH-PIN	TBFG_12770	TBFG_12771	–	–
Mycobacterium tuberculosis F11	vapBC/RHH-PIN	TBFG_12772	TBFG_12773	–	–
Mycobacterium tuberculosis F11	mazEF, ccd or relBE/RHH-MazF	TBFG_12814	TBFG_12815	–	–
Mycobacterium tuberculosis F11	-/RHH-GNAT	TBFG_12818	TBFG_12817	–	–
Mycobacterium tuberculosis F11	vapBC/PHD-PIN	TBFG_12844	TBFG_12845	–	–
Mycobacterium tuberculosis F11	relBE or parDE/PHD-RelE	TBFG_12882	TBFG_12881	–	–
Mycobacterium tuberculosis F11	vapBC/PHD-PIN	TBFG_13202	TBFG_13203	–	–
Mycobacterium tuberculosis F11	relBE/Xre-RelE	TBFG_13204	TBFG_13205	–	–
Mycobacterium tuberculosis F11	-/Xre-COG5654	TBFG_13209	TBFG_13208	–	–
Mycobacterium tuberculosis F11	vapBC/RHH-PIN	TBFG_13351	TBFG_13352	–	–
Mycobacterium tuberculosis F11	relBE or parDE/PHD-RelE	TBFG_13394	TBFG_13393	–	–
Mycobacterium tuberculosis F11	vapBC/PHD-PIN	TBFG_13418	TBFG_13419	–	–
Mycobacterium tuberculosis F11	-/MerR-PIN	TBFG_13781	TBFG_13782	–	–
Mycobacterium tuberculosis H37Ra	vapBC/RHH-PIN	MRA_0249	MRA_0248	–	–
Mycobacterium tuberculosis H37Ra	vapBC/RHH-PIN	MRA_0285	MRA_0286	–	–
Mycobacterium tuberculosis H37Ra	mazEF, ccd or relBE/RHH-MazF	MRA_0308	MRA_0307	–	–
Mycobacterium tuberculosis H37Ra	vapBC/RHH-PIN	MRA_0310	MRA_0309	–	–
Mycobacterium tuberculosis H37Ra	vapBC/RHH-PIN	MRA_0556	MRA_0557	–	–
Mycobacterium tuberculosis H37Ra	vapBC/PHD-PIN	MRA_0603	MRA_0604	–	–
Mycobacterium tuberculosis H37Ra	vapBC/AbrB-PIN	MRA_0606	MRA_0607	–	–
Mycobacterium tuberculosis H37Ra	vapBC/RHH-PIN	MRA_0616	MRA_0615	–	–
Mycobacterium tuberculosis H37Ra	vapBC/RHH-PIN	MRA_0633	MRA_0632	–	–
Mycobacterium tuberculosis H37Ra	vapBC/PHD-PIN	MRA_0636	MRA_0635	–	–
Mycobacterium tuberculosis H37Ra	vapBC/RHH-PIN	MRA_0667	MRA_0667A	–	–
Mycobacterium tuberculosis H37Ra	mazEF, ccd or relBE/RHH-MazF	MRA_0669	MRA_0670	–	–
Mycobacterium tuberculosis H37Ra	vapBC/RHH-PIN	MRA_0671	MRA_0672	–	–
Mycobacterium tuberculosis H37Ra	vapBC/RHH-PIN	MRA_0757	MRA_0756	–	–
Mycobacterium tuberculosis H37Ra	-/RHH-GNAT	MRA_0927	MRA_0926	–	–

Mycobacterium tuberculosis H37Ra	vapBC/RHH-PIN	MRA_1124	MRA_1123A	–	–
Mycobacterium tuberculosis H37Ra	vapBC/RHH-PIN	MRA_1251	MRA_1250	–	–
Mycobacterium tuberculosis H37Ra	relBE or parDE/PHD-RelE	MRA_1255	MRA_1255A	–	–
Mycobacterium tuberculosis H37Ra	vapBC/RHH-PIN	MRA_1406	MRA_1407	–	–
Mycobacterium tuberculosis H37Ra	vapBC/RHH-PIN	MRA_1573	MRA_1572	–	–
Mycobacterium tuberculosis H37Ra	vapBC/RHH-PIN	MRA_1730	MRA_1731	–	–
Mycobacterium tuberculosis H37Ra	vapBC/RHH-PIN	MRA_1752	MRA_1751	–	–
Mycobacterium tuberculosis H37Ra	vapBC/RHH-PIN	MRA_1849	MRA_1850	–	–
Mycobacterium tuberculosis H37Ra	vapBC/RHH-PIN	MRA_1963	MRA_1962	–	–
Mycobacterium tuberculosis H37Ra	relBE/Xre-RelE	MRA_1964	MRA_1965	–	–
Mycobacterium tuberculosis H37Ra	RelBE or parDE/RHH-RelE	MRA_1968	MRA_1969	–	–
Mycobacterium tuberculosis H37Ra	vapBC/PHD-PIN	MRA_1971	MRA_1972	–	–
Mycobacterium tuberculosis H37Ra	vapBC/RHH-PIN	MRA_1993	MRA_1994	–	–
Mycobacterium tuberculosis H37Ra	-/COG5642-COG5654	MRA_2003	MRA_2004	–	–
Mycobacterium tuberculosis H37Ra	mazEF, ccd or relBE/RHH-MazF	MRA_2006	MRA_2007	–	–
Mycobacterium tuberculosis H37Ra	vapBC/RHH-PIN	MRA_2026	MRA_2025	–	–
Mycobacterium tuberculosis H37Ra	-/COG2442-PIN	MRA_2035	MRA_2034	–	–
Mycobacterium tuberculosis H37Ra	relBE/Xre-RelE	MRA_2038	MRA_2037	–	–
Mycobacterium tuberculosis H37Ra	-/ArsR-COG3832	MRA_2050	MRA_2049	–	–
Mycobacterium tuberculosis H37Ra	mazEF, ccd or relBE/RHH-MazF	MRA_2077	MRA_2076	–	–
Mycobacterium tuberculosis H37Ra	vapBC/RHH-PIN	MRA_2117	MRA_2118	–	–
Mycobacterium tuberculosis H37Ra	vapBC/RHH-PIN	MRA_2520	MRA_2519	–	–
Mycobacterium tuberculosis H37Ra	vapBC/RHH-PIN	MRA_2554	MRA_2553	–	–
Mycobacterium tuberculosis H37Ra	vapBC/RHH-PIN	MRA_2557	MRA_2558	–	–
Mycobacterium tuberculosis H37Ra	vapBC/RHH-PIN	MRA_2574	MRA_2573	–	–
Mycobacterium tuberculosis H37Ra	vapBC/RHH-PIN	MRA_2576	MRA_2575	–	–
Mycobacterium tuberculosis H37Ra	vapBC/RHH-PIN	MRA_2578	MRA_2579	–	–
Mycobacterium tuberculosis H37Ra	vapBC/AbrB-PIN	MRA_2624	MRA_2623A	–	–
Mycobacterium tuberculosis H37Ra	vapBC/RHH-PIN	MRA_2630	MRA_2629A	–	–
Mycobacterium tuberculosis H37Ra	vapBC/RHH-PIN	MRA_2782	MRA_2783	–	–
Mycobacterium tuberculosis H37Ra	vapBC/RHH-PIN	MRA_2784	MRA_2785	–	–
Mycobacterium tuberculosis H37Ra	relBE or parDE/PHD-RelE	MRA_2891	MRA_2890	–	–
Mycobacterium tuberculosis H37Ra	vapBC/RHH-PIN	MRA_2896	MRA_2895A	–	–

Mycobacterium tuberculosis H37Ra	vapBC/PHD-PIN	MRA_3214	MRA_3215	–	–
Mycobacterium tuberculosis H37Ra	relBE/Xre-RelE	MRA_3216	MRA_3217	–	–
Mycobacterium tuberculosis H37Ra	-/Xre-COG5654	MRA_3221	MRA_3220	–	–
Mycobacterium tuberculosis H37Ra	vapBC/RHH-PIN	MRA_3362	MRA_3363	–	–
Mycobacterium tuberculosis H37Ra	relBE or parDE/PHD-RelE	MRA_3398	MRA_3397	–	–
Mycobacterium tuberculosis H37Ra	vapBC/PHD-PIN	MRA_3424	MRA_3425	–	–
Mycobacterium tuberculosis H37Ra	vapBC/PHD-PIN	MRA_3448	MRA_3447	–	–
Mycobacterium tuberculosis H37Ra	vapBC/RHH-PIN	MRA_3732	MRA_3733	–	–
Mycobacterium tuberculosis H37Ra	-/MerR-PIN	MRA_3787	MRA_3788	–	–
Mycobacterium tuberculosis H37Rv	-/-	Rv0059	Rv0060	–	–
Mycobacterium tuberculosis H37Rv	vapBC/RHH-PIN	Rv0065	Rv0064A	–	–
Mycobacterium tuberculosis H37Rv	vapBC/RHH-PIN	Rv0240	Rv0239	–	–
Mycobacterium tuberculosis H37Rv	mazEF, ccd or relBE/RHH-MazF	Rv0299	Rv0298	–	o
Mycobacterium tuberculosis H37Rv	vapBC/RHH-PIN	Rv0301	Rv0300	–	o
Mycobacterium tuberculosis H37Rv	-/RHH-PIN(toxin)	Rv0456a	Rv0456B	–	–
Mycobacterium tuberculosis H37Rv	vapBC/RHH-PIN	Rv0549c	Rv0550c	–	o
Mycobacterium tuberculosis H37Rv	vapBC/RHH-PIN	Rv0582	Rv0581	–	o
Mycobacterium tuberculosis H37Rv	vapBC/PHD-PIN	Rv0595c	Rv0596c	–	–
Mycobacterium tuberculosis H37Rv	vapBC/AbrB-PIN	Rv0598c	Rv0599c	–	–
Mycobacterium tuberculosis H37Rv	vapBC/RHH-PIN	Rv0609	Rv0608	–	o
Mycobacterium tuberculosis H37Rv	vapBC/-	Rv0617	Rv0616c	–	–
Mycobacterium tuberculosis H37Rv	vapBC/RHH-PIN	Rv0624	Rv0623	–	o
Mycobacterium tuberculosis H37Rv	vapBC/PHD-PIN	Rv0627	Rv0626	–	–
Mycobacterium tuberculosis H37Rv	vapBC/RHH-PIN	Rv0656c	Rv0657c	–	–
Mycobacterium tuberculosis H37Rv	mazEF/RHH-MazF	Rv0659c	Rv0660c	–	–
Mycobacterium tuberculosis H37Rv	vapBC/RHH-PIN	Rv0661c	Rv0662c	–	–
Mycobacterium tuberculosis H37Rv	vapBC/-	Rv0665	Rv0664	–	–
Mycobacterium tuberculosis H37Rv	vapBC/RHH-PIN	Rv0749	Rv0748	–	o
Mycobacterium tuberculosis H37Rv	-/-	Rv0836c	Rv0837c	–	–
Mycobacterium tuberculosis H37Rv	unclassified/-	Rv0910	Rv0909	–	o
Mycobacterium tuberculosis H37Rv	-/RHH-GNAT	Rv0919	Rv0918	–	–
Mycobacterium tuberculosis H37Rv	-/-	Rv0960	Rv0959A	–	–
Mycobacterium tuberculosis H37Rv	mazEF/-	Rv1102c	Rv1103c	–	o

Mycobacterium tuberculosis H37Rv	vapBC/RHH-PIN	Rv1114	Rv1113	–	o
Mycobacterium tuberculosis H37Rv	vapBC/RHH-PIN	Rv1242	Rv1241	–	o
Mycobacterium tuberculosis H37Rv	relBE/PHD-RelE	Rv1246c	Rv1247c	–	o
Mycobacterium tuberculosis H37Rv	vapBC/RHH-PIN	Rv1397c	Rv1398c	–	–
Mycobacterium tuberculosis H37Rv	mazEF/-	Rv1495	Rv1494	–	o
Mycobacterium tuberculosis H37Rv	unclassified/-	Rv1546	Rv1545	–	–
Mycobacterium tuberculosis H37Rv	vapBC/RHH-PIN	Rv1561	Rv1560	–	o
Mycobacterium tuberculosis H37Rv	vapBC/RHH-PIN	Rv1720c	Rv1721c	–	–
Mycobacterium tuberculosis H37Rv	vapBC/RHH-PIN	Rv1741	Rv1740	–	–
Mycobacterium tuberculosis H37Rv	vapBC/RHH-PIN	Rv1838c	Rv1839c	–	–
Mycobacterium tuberculosis H37Rv	mazEF/-	Rv1942c	Rv1943c	–	o
Mycobacterium tuberculosis H37Rv	vapBC/RHH-PIN	Rv1953	Rv1952	–	–
Mycobacterium tuberculosis H37Rv	higBA/Xre-RelE	Rv1955	Rv1956	–	o
Mycobacterium tuberculosis H37Rv	parDE/RHH-RelE	Rv1959c	Rv1960c	–	–
Mycobacterium tuberculosis H37Rv	-/RHH-RelE(toxin)	Rv1962c	Rv1963c	–	o
Mycobacterium tuberculosis H37Rv	vapBC/RHH-PIN	Rv1982c	Rv1982a	–	–
Mycobacterium tuberculosis H37Rv	-/COG5642-COG5654	Rv1989c	Rv1990c	–	–
Mycobacterium tuberculosis H37Rv	mazEF, ccd or relBE/RHH-MazF	Rv1991c	Rv1991a	–	o
Mycobacterium tuberculosis H37Rv	vapBC/RHH-PIN	Rv2010	Rv2009	–	o
Mycobacterium tuberculosis H37Rv	-/COG2442-PIN	Rv2019	Rv2018	–	–
Mycobacterium tuberculosis H37Rv	relBE/Xre-RelE	Rv2022c	Rv2021c	–	–
Mycobacterium tuberculosis H37Rv	-/ArsR-COG3832	Rv2035	Rv2034	–	–
Mycobacterium tuberculosis H37Rv	mazEF, ccd or relBE/RHH-MazF	Rv2063A	Rv2063	–	o
Mycobacterium tuberculosis H37Rv	vapBC/RHH-PIN	Rv2103c	Rv2104c	–	–
Mycobacterium tuberculosis H37Rv	-/-	Rv2142c	Rv2142A	–	o
Mycobacterium tuberculosis H37Rv	vapBC/PHD-PIN	Rv2231A	Rv2231B	–	–
Mycobacterium tuberculosis H37Rv	-/PHD-PIN(toxin)	Rv2274c	Rv2274A	–	–
Mycobacterium tuberculosis H37Rv	vapBC/RHH-PIN	Rv2494	Rv2493	–	–
Mycobacterium tuberculosis H37Rv	vapBC/RHH-PIN	Rv2527	Rv2526	–	–
Mycobacterium tuberculosis H37Rv	vapBC/RHH-PIN	Rv2530c	Rv2530A	–	o
Mycobacterium tuberculosis H37Rv	vapBC/RHH-PIN	Rv2546	Rv2545	–	–
Mycobacterium tuberculosis H37Rv	vapBC/RHH-PIN	Rv2548	Rv2547	–	o
Mycobacterium tuberculosis H37Rv	vapBC/RHH-PIN	Rv2549c	Rv2550c	–	–

Mycobacterium tuberculosis H37Rv	vapBC/AbrB-PIN	Rv2596	Rv2595	–	–
Mycobacterium tuberculosis H37Rv	vapBC/RHH-PIN	Rv2602	Rv2601A	–	o
Mycobacterium tuberculosis H37Rv	unclassified/-	Rv2653c	Rv2654c	–	o
Mycobacterium tuberculosis H37Rv	-/RHH-PIN(antitoxin)	Rv2697c	Rv3697A	–	–
Mycobacterium tuberculosis H37Rv	vapBC/RHH-PIN	Rv2757c	Rv2758c	–	o
Mycobacterium tuberculosis H37Rv	vapBC/RHH-PIN	Rv2759c	Rv2760c	–	–
Mycobacterium tuberculosis H37Rv	mazEF, ccd or relBE/RHH-MazF	Rv2801c	Rv2801A	–	o
Mycobacterium tuberculosis H37Rv	-/-	Rv2826c	Rv2827c	–	–
Mycobacterium tuberculosis H37Rv	vapBC/PHD-PIN	Rv2829c	Rv2830c	–	o
Mycobacterium tuberculosis H37Rv	-/-	Rv2863	Rv2862A	–	–
Mycobacterium tuberculosis H37Rv	relBE/PHD-RelE	Rv2866	Rv2865	–	o
Mycobacterium tuberculosis H37Rv	vapBC/RHH-PIN	Rv2872	Rv2871	–	o
Mycobacterium tuberculosis H37Rv	vapBC/PHD-PIN	Rv3180c	Rv3181c	–	–
Mycobacterium tuberculosis H37Rv	relBE/Xre-RelE	Rv3182	Rv3183	–	–
Mycobacterium tuberculosis H37Rv	-/Xre-COG5654	Rv3189	Rv3188	–	–
Mycobacterium tuberculosis H37Rv	vapBC/RHH-PIN	Rv3320c	Rv3321c	–	–
Mycobacterium tuberculosis H37Rv	relBE/PHD-RelE	Rv3358	Rv3357	–	o
Mycobacterium tuberculosis H37Rv	vapBC/PHD-PIN	Rv3384c	Rv3385c	–	o
Mycobacterium tuberculosis H37Rv	vapBC/PHD-PIN	Rv3408	Rv3407	–	o
Mycobacterium tuberculosis H37Rv	-/MerR-PIN	Rv3749c	Rv3750c	–	-
Bacillus anthracis str. Ames	mazEF/RHH-MazF	BA_0254	BA_0253	–	–
Bacillus anthracis str. Ames	-/Xre-Bro	BA_3827	BA_3828	–	–
Bacillus anthracis str. Ames	-/Xre-COG2856	BA_3830	BA_3829	–	–
Bacillus anthracis str. Ames	-/ArsR-COG3832	BA_4963	BA_4962	–	–
Bacillus anthracis str. Sterne	mazEF/RHH-MazF	BAS0240	BAS0239	–	o
Bacillus anthracis str. Sterne	-/Xre-Bro	BAS3543	BAS3544	–	–
Bacillus anthracis str. Sterne	-/Xre-COG2856	BAS3546	BAS3545	–	–
Bacillus anthracis str. Sterne	-/ArsR-COG3832	BAS4607	BAS4606	–	–
Bacillus cereus ATCC 10987	mazEF/RHH-MazF	BCE_0274	BCE_0273	–	–
Bacillus cereus ATCC 10987	-/Xre-COG2856	BCE_4623	BCE_4622	–	–
Bacillus cereus ATCC 10987	-/ArsR-COG3832	BCE_4853	BCE_4852	–	–
Bacillus cereus ATCC 10987	-/HEPN-MNT	BCE_A0102	BCE_A0103	–	–
Bacillus cereus ATCC 14579	mazEF/RHH-MazF	BC0266	BC0265	–	–

Bacillus cereus ATCC 14579	-/Xre-Bro	BC3702	BC3703	–	–
Bacillus cereus ATCC 14579	-/ArsR-COG3832	BC4709	BC4708	–	–
Bacillus cereus E33L	mazEF/RHH-MazF	BCZK0228	BCZK0227	–	–
Bacillus cereus E33L	-/Xre-Bro	BCZK3457	BCZK3458	–	–
Bacillus cereus E33L	-/Xre-COG2856	BCZK3460	BCZK3459	–	–
Bacillus cereus E33L	-/ArsR-COG3832	BCZK4461	BCZK4460	–	–
Bacillus cereus E33L	-/HEPN-MNT	pE33L466_0269	pE33L466_0270	–	–
Bacillus subtilis subsp. *subtilis* str. 168	bsrG-SR4/-	BSU21546	BSU_misc_RNA_77	–	o
Bacillus subtilis subsp. *subtilis* str. 168	rtbD-rtbE/-	BSU39300	BSU39290	–	o
Bacillus subtilis subsp. *subtilis* str. 168	mazEF/RHH-MazF	BSU04660	BSU04650	–	o
Bacillus subtilis subsp. *subtilis* str. 168	-/Xre-COG2856	BSU04810	BSU04820	–	–
Bacillus subtilis subsp. *subtilis* str. 168	SpoIISAB/-	BSU12830	BSU12820	–	o
Bacillus subtilis subsp. *subtilis* str. 168	-/Xre-GNAT	BSU19060	BSU19050	–	–

Reference

1. Shao, Y.; Harrison, E.M.; Bi, D.; Tai, C.; He, X.; Ou, H.Y.; Rajakumar, K.; Deng, Z. TADB: A web-based resource for Type 2 toxin–antitoxin loci in bacteria and archaea. *Nucleic Acids Res* **2011**, *39*, D606-D611.
